# Mechanistic origins of temperature scaling in the early embryonic cell cycle

**DOI:** 10.1101/2024.12.24.630245

**Published:** 2024-12-24

**Authors:** Jan Rombouts, Franco Tavella, Alexandra Vandervelde, Connie Phong, James E. Ferrell, Qiong Yang, Lendert Gelens

**Affiliations:** 1 Laboratory of Dynamics in Biological Systems, Department of Cellular and Molecular Medicine, KU Leuven, Herestraat, 49, Leuven, Belgium; 2 Cell Biology and Biophysics Unit and Developmental Biology Unit, European Molecular Biology Laboratory (EMBL), Heidelberg, Germany; 3 Department of Physics / Biophysics, University of Michigan, Ann Arbor, MI 48109, USA; 4 Department of Chemical and Systems Biology, Stanford University School of Medicine, Stanford, CA 94305-5174, USA

**Keywords:** Cell cycle, Cell-free extract, Xenopus laevis, Temperature, Thermal limits

## Abstract

Temperature profoundly impacts organismal physiology and ecological dynamics, particularly affecting ectothermic species and making them especially vulnerable to climate changes. Although complex physiological processes usually involve dozens of enzymes, empirically it is found that the rates of these processes often obey the Arrhenius equation, which was originally derived for single-enzyme-catalyzed reactions. Here we have examined the temperature scaling of the early embryonic cell cycle, with the goal of understanding why the Arrhenius equation approximately holds and why it breaks down at temperature extremes. Using experimental data from *Xenopus laevis, Xenopus tropicalis*, and *Danio rerio*, plus published data from *Caenorhabditis elegans, Caenorhabditis briggsae*, and *Drosophila melanogaster*, we find that the apparent activation energies $\left(E_{a}\;\text{values}\right)$ for the early embryonic cell cycle for diverse ectotherms are all similar, 76±9kJ/mol(mean±S.D.,n=6), which corresponds to a $Q_{10}$ value of 2.8±0.4(mean±S.D.,n=6). Using computational models, we find that the approximately Arrhenius scaling and the deviations from the Arrhenius relationship at high and low temperatures can be accounted for by biphasic temperature scaling in critical individual components of the cell cycle oscillator circuit, by imbalances in the $E_{a}$ values for different partially rate-determining enzymes, or by a combination of both. Experimental studies of cycling *Xenopus* extracts indicate that both of these mechanisms contribute to the general scaling of temperature, and in vitro studies of individual cell cycle regulators confirm that there is in fact a substantial imbalance in their $E_{a}$ values. These findings provide mechanistic insights into the dynamic interplay between temperature and complex biochemical processes, and into why biological systems fail at extreme temperatures.

## Introduction

Living organisms are continually influenced by their environment, and embryos are particularly sensitive to environmental changes. Even subtle perturbations during critical developmental windows can significantly impact embryo viability, as well as embryonic and post-embryonic performance (1). For example, changes in temperature can profoundly influence embryonic development, influencing the speeds of biochemical reactions and affecting the overall physiology, behavior, and fitness of the organism (2–6).

Ectotherms, in particular, rely critically on the ambient temperature as they have minimal ability to generate heat internally. Each ectothermic species has a specific temperature range associated with its geographic distribution on the planet (7, 8). While adult stages of ectotherms can adopt various physiological and behavioral strategies to maintain optimal temperatures, such as taking shelter when it is too hot (6), embryos possess limited mechanisms to cope with environmental challenges, making them the most vulnerable life stage to environmental stress (9, 10). Temperature plays a pivotal role in determining the fertilization rate of eggs, the growth and survival of embryos, and in certain cases, even the gender of offspring, as observed in many turtle species and all crocodiles (11). Assessing the thermal impact on and sensitivity of embryonic development across a range of temperatures provides essential insights into species responses and vulnerability to the challenges posed by global warming (8, 9, 12). Indeed, the impact of global warming is already evident in certain sea turtle species, where a diminishing number of male offspring is observed (13).

But even without this shifting landscape and the challenges of their changing ecosystems, ectotherms face the daunting challenge of needing to have their biochemistry function reliably over a wide range of temperatures. Given that complex metabolic networks, signaling systems, and developmental processes may involve dozens of enzymes, the question arises as to how much variation in the individual enzymes’ temperature scaling can be tolerated before the system fails.

The influence of temperature on physiology and development has been a subject of study for over a century (3, 4, 14). The relationship between temperature and the speed of many diverse biological processes is often well approximated by the Arrhenius equation (15–22). Originally formulated for simple, one-step chemical reactions, the Arrhenius equation describes the rate of a chemical reaction (k) as a function of the absolute temperature (T):

(1) $$k\left(T\right)=Ae^{\frac{-E_{a}}{RT}},$$

where $E_{a}$ denotes a temperature-independent activation energy, R is the universal gas constant, and the pre-exponential factor A sets the maximal reaction rate at high temperatures. While derivable for elementary chemical reactions from thermodynamic principles, this equation is considered an empirical law applicable to various physiological rates (16). However, deviations from this Arrhenius response are consistently observed at higher temperatures (18, 22–25). The decline is often attributed to the heat denaturation of some critical enzyme (18, 25–27), and the trade-off between increasing reaction rate and increasing denaturation yields an optimal temperature. Moreover, the thermal response of processes at the cellular level is influenced not only by the reactions of enzymes but also by active cellular responses to temperature changes. For instance, in response to stress, cells may up-regulate heat shock proteins, aiding in protein refolding (28). In addition, budding yeast can up-regulate the production of viscogens like trehalose and glycogen to help maintain normal diffusion kinetics at elevated temperature (29).

Recently, the topic of biological temperature scaling has garnered renewed interest, thanks to the ability to obtain accurate, high resolution data through time-lapse microscopy. This approach has provided fresh insights into early development in several model systems (30–33). Overall these findings reaffirm the utility of the Arrhenius equation as a reliable approximate description of the temperature scaling of embryonic development. This was particularly clear in studies of the timing of the first embryonic cell cycle in *C. elegans* and *C. briggsae*, two closely related nematodes. In both species, the duration of the cell cycle as a function of temperature precisely agreed with the Arrhenius equation over a broad temperature range, with some deviation then occurring when the embryos were close to their maximum tolerated temperatures (31). The two nematodes also were found to have almost identical Arrhenius energies ($E_{a}$ values), which raises the possibility that the activation energies of cell cycle regulators may be evolutionarily constrained to a single standard value (31).

*Xenopus laevis* extracts and embryos have proven to be powerful systems for the quantitative analysis of the early embryonic cell cycle. The cell cycle can be experimentally studied both in vivo and in extracts, and extracts can be manipulated and observed in ways that are difficult with intact embryos. In addition, much is already known about cell cycle biochemistry in this system, providing a rich and highly quantitative context for further studies. And finally, the dynamics of the cell cycle can be successfully reproduced with relatively simple mathematical models that can add depth to the understanding of experimental findings. Crapse and colleagues have begun to examine how the *Xenopus* embryonic cell cycle is affected by temperature, and they have found some striking similarities to the behaviors seen in *C. elegans* and *C. briggsae*: the cell cycle period obeys the Arrhenius equation at least approximately, and the measured $E_{a}$ value for the cell cycle is similar to those in the two nematodes (32) .

Here we have leveraged the *Xenopus* system to address several outstanding questions on the principles of biological temperature scaling. First, we compared the *Xenopus laevis* temperature scaling to that in two other ectothermic vertebrate model systems, *Xenopus tropicalis* and *Danio rerio*, and compared the findings to previously reported data from the invertebrates *C. elegans*, *C. briggsae*, and Drosophila melanogaster. Second, we asked how well the temperature scaling is described by the Arrhenius equation, and based on ordinary differential equation modeling of the cell cycle, under what circumstances would the cell cycle be expected to exhibit Arrhenius scaling, and under what circumstances would it be expected to deviate. Finally, we asked how the different individual phases of the cell cycle vary with temperature, and found that interphase and mitosis scale differently and that this difference can be accounted for by the in vitro thermal properties of key cell cycle regulators. These studies provide insight into the principles that allow ectotherms to tolerate a range of temperatures, and suggest mechanisms for why the cell cycle oscillator fails at temperature extremes.

## Results

### Temperature scaling of cell division timing in the *Xenopus laevis* embryo.

**Fig. 1:**
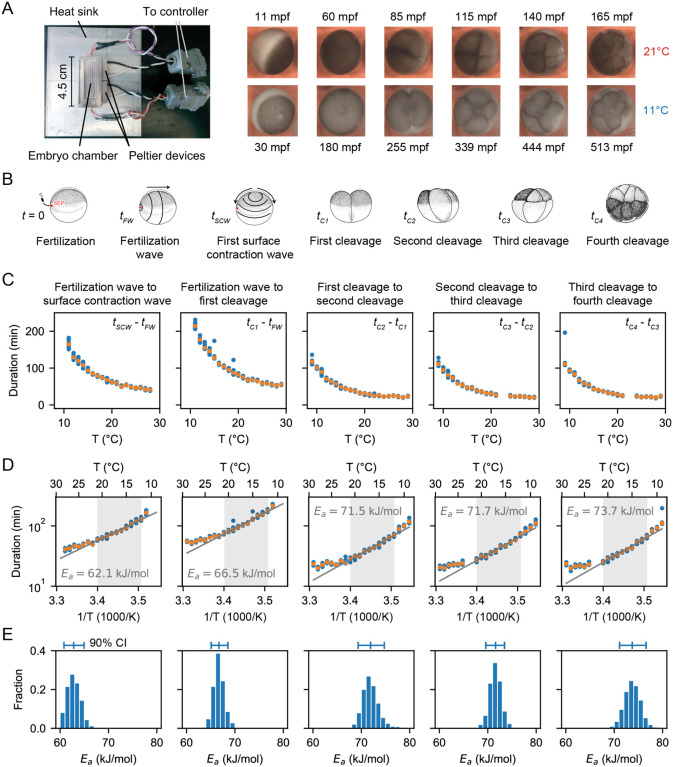
Cell division timing in early Xenopus laevis embryos does not scale Arrhenius. A. *Xenopus laevis* embryonic development was imaged in a temperature-controlled chamber introduced in Ref. (34). mpf is minutes post-fertilization. B. Different early developmental events were visually identified. Sperm entry point (SEP) denotes the sperm entry point. Adapted from (87). C. Duration of several early developmental periods in function of temperature in the range [$T_{\text{min}}=9^{\circ }C,\;T_{\text{max}}=29^{\circ }C$]. D. An Arrhenius fit is shown for the values between 12°C and 21°C, with the apparent activation energy indicated. E. Bootstrapping provides a probability distribution for the apparent activation energies. The mean and 90% confidence interval (CI) are also indicated.

Building on the initial studies of Crapse and colleagues (32), we set out to measure the temperature dependence of the timing of several early cell cycle events in the developing early *Xenopus laevis* embryo (Figure 1A–B). *Xenopus laevis* eggs were fertilized and imaged in a temperature-controlled chamber (first described in Ref. (34)) by time-lapse microscopy (Fig. 1A). We then analyzed the movies (Supplemental Videos 1–2) to visually identify various early developmental events (Fig. 1B). First, we scored the start of the fertilization wave, a ripple in the egg’s cortex that quickly spreads from the sperm entry point across the egg (at time $t_{\text{FW}}$ after fertilization). This wave is due to a trigger wave of elevated intracellular calcium, and it contributes to the block to polyspermy and coordinates the start of the cell cycle (35). Next, we measured the start of the first surface contraction wave, which emanates from the animal pole and travels toward the vegetal pole (36, 37) (at time $t_{\text{SCW}}$ after fertilization). This wave marks mitotic entry and has been argued to be caused by the interaction of a spherical wave of Cdk1 activation originating at the nucleus (38–40) with the cortical cytoskeleton (41–43). Finally, we assessed the cleavages that complete each of the first four cell cycles. The first cleavage begins about 95 min after fertilization at 18°C, and the next several cycles occur every 35 min thereafter (44–46). For multicellular embryos, we took the time at which the earliest cell began to divide to be the cleavage time, but note that within an embryo, these cell divisions were nearly synchronous. The timing of all of these events was recorded for about 10 different embryos at each temperature.

For temperatures between 10°C and 28°C, fertilized embryos reliably cycled. Just above (to 29°C) and below (to 9°C) this range there were general still a few cycles, and these data are included in Fig. 1. We calculated the time intervals between various events and plotted how they vary with temperature (Fig. 1C–D). Note that the Arrhenius equation (Eq. (2)) can be rearranged to relate the duration of a process, Δt, to the absolute temperature:

(2) $$\Delta t[T]=\frac{1}{k[T]}=\frac{1}{A}e^{\frac{E_{a}}{RT}},$$

(3) $$\text{ln}\Delta t\left[T\right]=\text{ln}\frac{1}{A}+\frac{E_{a}}{R}\frac{1}{T}.$$

Thus we replotted the data as lnΔt versus 1/T (Fig. 1D). Over a range of 12°C to 21°C, the data were well-approximated by Eq. (3), and from the fitted slopes we extracted Arrhenius energies $\left(E_{a}\right)$. The earliest measured event, the interval between the start of the fertilization wave and the start of the first surface contraction wave, was found to correspond to an Arrhenius energy of 62±3 kJ/mol, in agreement with previous findings (32). This Arrhenius energy is well within the range said to be typical for enzymes, 20 – 100 kJ/mol (47–49). Above and below the 12 – 21°C temperature range, the durations deviated from the linear relationship, with longer than expected durations seen at both ends of the range (Fig. 1D).

Similar response curve shapes and deviations from linearity were found for the Arrhenius plots of the later cell cycle periods (Fig. 1D). The apparent activation energy progressively increased with each cell cycle (Fig. 1D). The statistical significance of the activation energy differences was confirmed using a bootstrapping method, providing a probability distribution for the apparent activation energies (Fig. 1E, Fig. S1A, Supplementary Note 2).

Finally, we calculated the deviation of the data from this fitted function as represented by the mean square error (MSE) over the whole measured temperature range (9°C to 29°C) and over the Arrhenius range (12°C to 21°C). As expected, the mean square error was much higher over the whole temperature range than within the selected range, indicating that the Arrhenius equation does not fit the data well over the whole measured data range (Fig. S1B).

### Diverse ectothermic species yield similar temperature scaling.

**Fig. 2:**
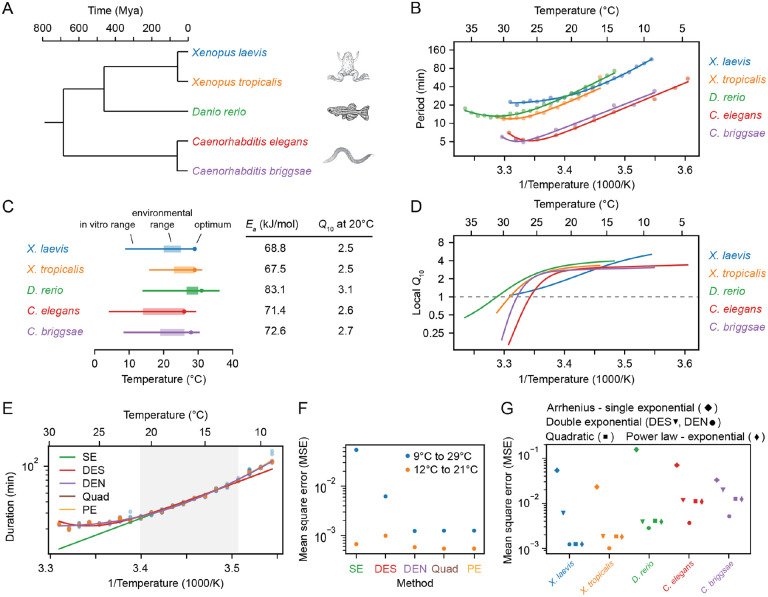
Cell cleavage period scales in a similar non-Arrhenius way across different early ectothermic embryos. A. We examined the timing of the early cell cycles in 5 different species: *C. elegans* and *C. briggsae* (from (31)), D. rerio (this work), *X. tropicalis* and *X. laevis* (this work). The three vertebrates and the two nematodes span a broad range in evolution. B. Median cleavage period in function of temperature for the second to fourth cell cycle (all pooled) for the 5 different species. Optimal fits using a double exponential (DE) function are overlayed. C. The in vivo range of viable early cell cycles in the different species, including their thermal limits and optimal temperature at which they reach a minimum cell cycle period. Their corresponding apparent activation energies and $Q_{10}$ at 20°C are shown in the table. Additionally the environmental range is indicated for all five organisms (88–91). D. Using the best DE fit, the local $Q_{10}$ value is plotted in function of temperature. E. The median cleavage period in function of temperature for *X. laevis* is fitted using different functional forms: single exponential Arrhenius (SE), double exponential (nonlinear fit: DEN, sequential linear fit: DES), and a power law - exponential (PE) function. F. The goodness of fit (using mean square error, MSE on the logarithms of the periods) of the alternative functional forms to the experimental data for *X. laevis* in two different temperature regions: 12–21°C and 9–29°C. G. Goodness of fit, similar as in panel F, but now for all different species over their whole measured temperature range.

Next we examined the timing of the early cell cycles in two additional vertebrate model organisms, the frog *X. tropicalis* (Supplemental Videos 3–4) and the zebrafish *D. rerio* (Supplemental Videos 5–6). The three vertebrates and the two nematodes span a broad range in evolution (Fig. 2A).

The period of the early embryonic cell cycle as a function of temperature for all five organisms is shown in Fig. 2B. For simplicity, here we have pooled data for the durations of the second to fourth cell cycles. In all cases, the early embryonic cell cycle could proceed over a 15–25° range of temperatures. In general, the five organisms showed reasonable agreement with the Arrhenius equation, especially toward the lower end of their temperature ranges (Fig. 2B). *Xenopus laevis* was something of an outlier in this regard; its Arrhenius plot is bowed throughout the temperature range (Fig. 2B, blue). The apparent Arrhenius energies—the slopes of the Arrhenius plots—were quite similar, ranging between 68 and 83 kJ/mol, or 73 ± 6 kJ/mol (mean ± S.D., n = 5).

As might be expected, the nominal ambient environmental temperatures for all five organisms fell within the range found to be compatible with cell cycle oscillations (Fig. 2C). The temperature ranges for Xenopus tropicalis and *Danio rerio* were shifted toward higher temperatures compared to *Xenopus laevis*, reflecting the fact that the former two evolved in and live in warmer regions (Fig. 2C, orange and green vs. blue). A similar shift in the viable temperature range has been noted for the nematode worms *C. elegans* and *C. briggsae* (31), replotted here in red and purple. In all cases, the maximum temperature compatible with cycling was closer to the nominal environmental temperature range than the minimum temperature was (Fig. 2C).

For four of the five organisms (*C. elegans, C. briggsae, D, rerio*, and *X. tropicalis*) there was sufficient upward deflection of the temperature curves toward the high end of the temperature range to define an optimal growth temperature corresponding to a minimal cell cycle duration (Fig. 2C). For *Xenopus laevis*, the fastest cell cycles were found at the highest temperature compatible with viability (Fig. 2C). In all cases the optimal temperature was within a few degrees of the maximal permissible temperature $T_{\text{max}}$ (Fig. 2C). The optimal temperatures were generally somewhat higher than the typical environmental temperature ranges (Fig. 2C). This may reflect a trade-off between maximal speed at higher temperatures and maximal safety margins in the middle of the operating temperature range. Note that at the temperature optima, the slopes of the Arrhenius plots are zero. The curves are also shallow at the optima, which means that changes of several degrees produce little changes in the cell cycle period. The period can be regarded as temperature-invariant or temperature-compensated in this regime.

Data are also available for the temperature scaling of various embryonic processes in *Drosphila melanogaster*. Extensive data are available for the timing of the 13th cycle (32), and these data are replotted in Fig. S2A. Like the *Xenopus laevis* Arrhenius plot, the *Drosophila* plot is bowed throughout the temperature range, and, at the cold end of the temperature range, it yielded an Arrhenius energy of ≈ 109 kJ/mol (Fig. S2B). Note, however, that this cycle differs from the earlier *Drosophila* cycles and the other embryonic cycles examined here in possessing a G2 phase, and so it may not scale the way the earlier cycles do (50). Some data are available for the 11th nuclear cycle (NC11) in the syncytial *Drosophila* embryo (Fig. S3) (33); this cycle is more similar to the other organisms’ cycles analyzed here in its lack of gap phases. From the published data, we calculated an Arrhenius energy of 91 ± 8 kJ/mol for the duration of NC11 (mean ± std. err. of the fitted value (Fig. S2A), about 25% higher than the energies calculated for the early embryonic cycles of the other 5 model organisms. Taken together, the six organisms yielded an average Arrhenius energy for the early embryonic cell cycles of 76 ± 9 kJ/mol (mean ± std. dev.). Although the nominal periods of the cell cycles varied greatly, from about 5 min for *C. elegans* and *C. briggsae* to 25 min for *X. laevis* at room temperature, the temperature scaling factors for these organisms varied by only about 12%.

### $Q_{10}$ values vary continuously with temperature from about 1 to 4.

The effect of temperature on a biological process is often expressed as a $Q_{10}$ value, which quantifies the fold-change in the rate of a process across a temperature range of 10°C (51):

(4) $$Q_{10}[T]=\frac{k[T+10]}{k[T]}$$

For a process described by the Arrhenius equation, the $Q_{10}$ value reduces to:

(5) $$Q_{10}[T]=e^{\frac{E_{a}}{RT}-\frac{E_{a}}{R(T+10)}}.$$

For the three vertebrates and the three invertebrates, the $Q_{10}$ values were very similar; if we take $T=20^{\circ }C$ and use the $E_{a}$ values from linear fits over the Arrhenius temperature ranges, the $Q_{10}$ values ranged from 2.5 to 3.4, with a mean of 2.8 and a standard deviation of 0.4 (Fig. 2C). This indicates that the timing of the early embryonic cell cycle scales very similarly with temperature for diverse organisms.

Even for a process that strictly obeys the Arrhenius equation, the $Q_{10}$ value varies with temperature, and for non-Arrhenius processes, the variation can be substantial. Thus the common practice of providing a single $Q_{10}$ value to describe the temperature dependence of a process is perhaps not optimal. For this reason, a local $Q_{10}$ value, defined as:

(6) $$Q_{10}\left[T\right]=e^{10\frac{k^{'}\left[T\right]}{k\left[T\right]}},$$

where $k^{'}(T)$ is $\frac{dk(T)}{dT}$, can be calculated and plotted (see Supplementary Note 1). If the local $Q_{10}$ value is greater than 1, the process is speeding up with increasing temperature and the Arrhenius slope is positive; if it is less than 1, the process is slowing down with temperature and the Arrhenius slope is negative. As shown in Fig. 2D, for all organisms except *Xenopus laevis*, the local $Q_{10}$ value plateaued at approximately 2.8 at the low end of the temperature range, and fell to less than 1 at high temperatures. For *Xenopus laevis*, the local $Q_{10}$ value varied continuously from about 1 to 4 throughout the temperature range (Fig. 2D, Fig. S2D).

### Different generalizations of the Arrhenius equation can describe the observed temperature scaling.

Although the Arrhenius relationship approximated the temperature scaling data, in all cases there was some deviation. Several generalizations of the Arrhenius equation, based on distinct physical assumptions, have been previously suggested (32, 52–56); here we have evaluated how well three of these generalized Arrhenius equations fit the data:

The *Double Exponential* (DE) function, which contains two exponential functions and four free parameters:

(7) $$k(T)=A_{1}e^{\frac{-E_{a1}}{RT}}+A_{2}e^{\frac{-E_{a2}}{RT}}.$$

A similar double exponential function has been used to fit the growth rate of *E. coli* based on the transition state theory of Eyring (52), where the additional exponential term accounts for reversible protein denaturation at high temperature (53), and it has already been used to fit the temperature scaling of the early cell divisions in the nematode worms *C. elegans* and *C. briggsae* (31). We employed two distinct approaches to fit the data to a DE function. Firstly, we adopted the sequential fitting method, denoted as DES for double exponential sequential, as outlined in (31). This method involves two consecutive fits: initially, a fit of the “Arrhenius interval” at lower temperatures using a single exponential ($E_{a1},A_{1}$) is performed. Subsequently, with these parameters held fixed, another single exponential fit is conducted to determine the values of ($E_{a2},A_{2}$). Secondly, we executed a full nonlinear fit, denoted as DEN for double exponential nonlinear, aiming to directly ascertain all four optimal parameters ($E_{a1},A_{1},E_{a2},A_{2}$). This approach is computationally more intensive compared to the sequential fitting method.

The *Quadratic Exponential* (QE) function, which contains three free parameters, has been derived in the limit of large system size and if the biochemical control network has a bias towards a target state (56). This function has recently been shown to provide a good description of the temperature scaling of early developmental events in *X. laevis* and in *D. melanogaster* (32, 56):

(8) $$k(T)=Ae^{\frac{-E_{a}}{R}\left(\frac{1}{T}+\frac{B}{T^{2}}\right)}.$$

The *Power law - Exponential* (PE) function, which is based on Eyring-Evans-Polanyi (54) transition state theory and has been put forward as a general theory for temperature dependence in biology (55):

(9) $$k\left(T\right)=AT^{B}e^{\frac{-E_{a}}{RT}}.$$

These three alternative functional forms all provided a much better fit to the experimentally measured data than did the regular Arrhenius relationship, and the double exponential nonlinear fit (DEN) performed somewhat better than the double exponential sequential fit (DES) (Fig. 2E–G). Moveover, the three alternative forms were about equally good at accounting for the experimental data. This means that one cannot infer which set of underlying mechanistic assumptions is most consistent with the experimental results.

### A simple relaxation oscillator model for the early embryonic cell cycle can account for approximate Arrhenius scaling as well as the observed deviations from Arrhenius scaling.

We next took an alternative approach to the question of why cell cycle periods at least approximately obey the Arrhenius equation, and why they sometimes deviate from Arrhenius scaling. The approach was to use a computational model of the embryonic cell cycle oscillator and see how the modeled period would be expected to vary with temperature, using either single or double exponential equations for the individual enzymes’ temperature scaling. This allowed us to examine how systems-level properties of the cell cycle oscillator circuit, rather than just variations from the Arrhenius relationship in the behaviors of individual enzymes, might be expected to affect the temperature scaling of the oscillations.

The cell cycle regulatory network consists of many complex interactions involving dozens of species, which makes it extremely challenging to construct a complete mathematical model, let alone study and interpret the influence of temperature on the cell cycle. The early embryonic cell cycle of insects, worms, amphibians and fish is, however, much simpler: the cycle consists of a rapid alternating sequence of synthesis (S) phase and mitotic (M) phase, without checkpoints and without G1 and G2 gap phases. Transcription is negligible at this point in embryogenesis, and the number of protein species involved is smaller than in the somatic cell cycle. As a result, simpler mathematical models can be constructed. This greatly simplifies the analysis of temperature scaling.

**Fig. 3:**
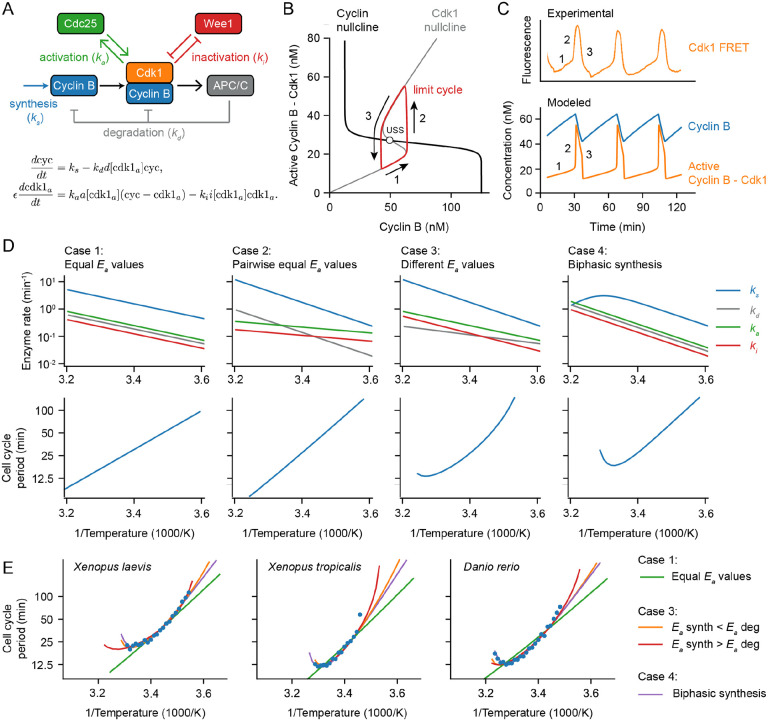
A simple relaxation oscillator model for the early embryonic cell cycle can reproduce the observed non-Arrhenius scaling. A. Sketch of key reactions in the early cell cycle regulatory network. B-C. Phase plane representation (B) and time series (C) of cell cycle oscillations in Eq. (23). D. Scenarios showing how different temperature scaling of cell cycle regulatory processes can lead to Arrhenius scaling and/or thermal limits in the scaling of the cell cycle period. E. Best fits of models presented in D to the measured data for the early cell cycle duration for *X. laevis*, *X. tropicalis* and *D. rerio* shown in Fig. 2. Case 4, introducing a thermal limits in a key cell cycle process allows to describe the data. For case 3, the apparent activation energies for $k_{s}$ and $k_{d}$ need to be different to fit the data well. E. Error of simulated data (case 3) in describing the experimental data in D (using the rMSE). The influence of apparent activation energies for $k_{s}$ and $k_{d}$ are studied. For parameter values and more details about the model, see Supplementary Note 3.

At the heart of the early embryonic cell cycle lies the protein complex cyclin B – Cdk1, consisting of the protein cyclin B and the cyclin-dependent-kinase Cdk1 (see Fig. 3A), plus a phosphoepitope-binding subunit, Suc1/Cks, that is not separately considered here. When this protein kinase complex is enzymatically active, it phosphorylates hundreds of other proteins, bringing about the entry of the cell into mitosis (57, 58). The oscillations in Cdk1 activity result from three interlinked processes: (1) cyclin B synthesis, which dominates in interphase and causes Cdk1 activity to gradually rise; (2) the flipping of a bistable switch, due in this model to the Cdk1-Cdc25 positive feedback loop and the Cdk1-Wee1 double negative feedback loop, which results in an abrupt rise in Cdk1 activity; and (3) cyclin B degradation by the APC/C and the proteosome, which dominates during M-phase and causes Cdk1 activity to fall (Fig. 3B). This type of oscillator, consisting of a rapid bistable switch plus a negative feedback loop, is referred to as a relaxation oscillator (59–61). Relaxation oscillators are common in biology, and all relaxation oscillators share similar qualitative behavior, irrespective of the exact molecular details: there is a slow ramp up in activity, which then triggers an abrupt burst in activity through the positive feedback loop(s), and finally the negative feedback restores the system back to its low activity state. These three distinct phases can be distinguished in experimental data on the activity of Cdk1 as a function of time in the early embryonic cell cycle (Fig. 3C; see also below).

Note that other enzymes, processes, and feedback loops contribute to the cell cycle oscillator, most notably the Greatwall/PP2A-B55 system, whose regulation is critical for the switches into and out of M-phase (61–66). Nevertheless, a simple system of two ordinary differential equations (ODEs) is sufficient to capture the dynamics of Cdk1 activation and inactivation (67):

(10) $$\frac{d\text{cyc}[t]}{dt}=k_{s}-k_{d}\text{AP}{\text{C}}_{a}[t]\text{cyc}[t],$$

(11) $$\epsilon \frac{d\text{cdk}1_{a}[t]}{dt}=k_{a}\text{Cdc}25_{a}[t]\left(\text{cyc}[t]-\text{cdk}1_{a}[t]\right)-k_{i}\text{Wee}1_{a}[t]\text{cdk}1_{a}[t].$$

The first equation describes how cyclin B (cyc) is synthesized throughout the cell cycle at a rate $k_{s}$ (nM/min) and how it is degraded at a rate $k_{d}$ (1/min) by the proteasome after ubiquitination by active APC/C (${\text{APC}}_{a}[t]$). The second equation describes the conversion of cyclin B-Cdk1 complexes between an inactive form and an active form by Cdc25 ($\text{Cdc}25_{a}[t]$) and Wee1 ($\text{Wee}1_{a}[t]$). If we assume that the Cdk1-mediated phosphorylation reactions that regulate APC/C, Wee1, and Cdc25 are essentially instantaneous, we can eliminate three of the time-dependent variables from the right hand side of the ODEs:

(12) $$\frac{d\text{cyc}[\text{t}]}{dt}=k_{s}-k_{d}d\left[\text{cdk}1_{a}\right]\text{cyc}[t],$$

(13) $$\epsilon \frac{d\text{cdk}1_{a}[t]}{dt}=k_{a}a\left[\text{cdk}1_{a}\right]\left(\text{cyc}[t]-\text{cdk}1_{a}[t]\right)-k_{i}i\left[\text{cdk}1_{a}\right]\text{cdk}1_{a}[t].$$

The terms $d\left[\text{cdk}1_{a}\right],a\left[\text{cdk}1_{a}\right]$, and $i\left[{\text{cdk}}_{a}\right]$ are assumed to be Hill functions of the instantaneous values of $\text{cdk}1_{a}$, and were parameterized based on experimental measurements of steady-state responses in *Xenopus laevis* extracts (67–69). We thus have two ODEs in two time-dependent variables, cyc[t] and ${\text{cdk}1}_{a}[t]$, and five parameters that define the speeds of cyclin synthesis $\left(k_{s}\right)$, cyclin degradation $\left(k_{d}\right)$, Cdk1 activation by Cdc25 $\left(k_{a}\right)$, and Cdk1 inactivation by Wee1 $\left(k_{i}\right)$, as well as the relative time scales of cyclin synthesis and degradation versus Cdk1 activation and inactivation (ϵ). Even though this simplified model omits Greatwall/PP2A-B55 regulation and a number of other interesting aspects of *Xenopus* cell cycle regulation, it nevertheless captures the dynamics of Cdk1 activation and inactivation well and has been used to successfully describe various aspects of cell cycle oscillations (34, 67, 70). For this reason it seemed like a good starting point for understanding how the output of the cell cycle oscillator circuit would be expected to scale with temperature.

Using experimentally motivated parameters (67), the model (Eqs. (12)–(13)) reproduced cell cycle oscillations with a realistic period of approximately 30 min (Fig. 3C). These oscillations manifested as a closed trajectory, a limit cycle, in the (cyc, $\text{cdk}1_{a}$) phase plane (Fig. 3B, red), which orbits around an unstable steady state (Fig. 3B, USS) at the intersection of the system’s two nullclines (Fig. 3B). When the time scale for Cdk1 activation/inactivation is fast relative to the time scale of cyclin synthesis and degradation (ϵ≪1), typical relaxation oscillations occur: in interphase, the orbit slowly creeps up the low Cdk1 activity portion of the S-shaped nullcline (Fig. 3B, denoted 1), then abruptly jumps up to the high Cdk1 activity portion of the same nullcline (Fig. 3B, denoted 2), crawls down the nullcline due to active APC/C (Fig. 3B, denoted 3), and abruptly falls back down to the lower portion of the nullcline to begin the cycle again. The result is sawtooth-shaped oscillations in cyclin B levels and periodic bursts of Cdk1 activity that resemble experimentally-measured Cdk1 activities (Fig. 3C).

Next we examined how making the model’s parameters temperature-dependent affected the persistence and period of the oscillations over a range of temperatures. We started by assuming Arrhenius scaling for the four key rates in the oscillator model: $k_{s},k_{d},k_{a}$, and $k_{i}$. As expected, when all of the apparent activation energies were assumed to be equal, the oscillation period obeyed the Arrhenius equation with the same activation energy (Case 1 in Fig. 3D). Similarly, if the activation energies for cyclin synthesis and degradation were assumed to be equal, and the energies for Cdk1 activation and inactivation were assumed to be different from those but equal to each other, the period also scaled in an Arrhenius fashion (Case 2 in Fig. 3D), with $E_{a}$ equal to that of cyclin synthesis and degradation. This Arrhenius scaling arises out of three properties of the model: (1) the cyclin nullcline’s position depends solely on the ratio $k_{s}/k_{d}$, and these two parameters were assumed to scale identically with temperature; (2) the location of the S-shaped Cdk1 nullcline depends upon the ratio $k_{a}/k_{i}$, which likewise was assumed to scale identically with temperature; and (3) as long as ϵ is very small, the cell cycle period is determined only by the rates of cyclin synthesis and degradation, which scale identically with temperature.

However, if the four kinetic parameters were not constrained to scale identically (Case 1) or pair-wise identically (Case 2) with temperature, the results were more like what is seen experimentally. This is shown as Fig. 3D, Case 3. The Arrhenius plot was bowed concave up, instead of being straight, and oscillations ceased if the temperature was too high or too low. The cessation of oscillations can be rationalized from the positions of the nullclines in the phase plane. If the $E_{a}$ value for cyclin synthesis is smaller than that for cyclin degradation, the ratio $k_{s}/k_{d}$ decreases as temperature rises. With increasing temperature, the cyclin nullcline shifts down until it no longer intersects the middle portion of the S-shaped Cdk1 nullcline. At this point, the steady state becomes stable and oscillations cease, leaving the system in an interphase-like steady state with low Cdk1 activity (Fig. S4). Conversely, if cyclin synthesis scales more strongly with temperature than degradation does, the cyclin nullcline shifts upward, leading to a stable M-phase-like steady state with high Cdk1 activity. Thus, if the temperature scaling of cyclin synthesis and degradation differ, at extremes of the temperature range the oscillator will fail, and in between the extremes the cell cycle period would be expected to deviate from the Arrhenius relationship. This could provide an explanation for the temperature scaling observed experimentally (Fig. 1).

An alternative assumption could also explain the experimental results. As shown in Fig. 3D, Case 4, if at least one process shows a biphasic dependence of rate on temperature, perhaps due to enzyme denaturation at high temperatures, the result will be a bowed Arrhenius plot and a high temperature limit to oscillations. As an example, here we have assumed a biphasic dependence of cyclin synthesis on temperature. Thus, in principle it seemed like either variation in the individual enzymes’ Arrhenius energies (Case 3), or denaturation at high temperatures (Case 4), or both, could account for the observed temperature scaling of the early embryonic cell cycle.

To test this hypothesis further, we asked how well model Cases 3 and 4 could replicate the observed cell cycle duration scaling in early frog and zebrafish embryos. We adjusted the apparent activation energies of cyclin synthesis and degradation, and, for simplicity, kept the $E_{a}$ values for Cdk1 activation and inactivation constant. Through minimizing the error between simulations and data across the oscillation range, employing the mean sum of squares on logarithms of periods, we obtained optimal fits. Fig. 3E displays these fits across various model scenarios introduced in Fig. 3D. Although perfect Arrhenius scaling (Cases 1 and 2) did not align well with the data, model Cases 3 and 4 approximated the measured data well. Notably, the experimental data could be accounted for by assuming that cyclin synthesis scaled either more strongly or more weakly with temperature than did cyclin degradation.

To further reinforce these findings, we conducted an exhaustive parameter scan over the activation energies of all four key rates in the oscillator model using a fitting algorithm. We employed the approximate Bayesian computation method, implemented in Python using pyABC, for sequential Monte Carlo sampling of parameter sets, gradually improving fits to the data (for details, see Supplementary note C and Fig. S5). This broader analysis underscored that optimal fits occurred when there was a distinct difference in apparent activation energies between cyclin synthesis and degradation, while the activation energies of Cdk1 activation and inactivation remained similar (Fig. S5). Moreover, the quantification of fitting errors revealed that it is most probable that the experimental data is due to cyclin synthesis being more sensitive to temperature changes than cyclin degradation $\left(E_{a}\left(k_{s}\right)>E_{a}\left(k_{d}\right)\right)$.

Next we asked whether these results were specific to this one particular cell cycle model. We constructed a five-ODE, mass action-based model that included the interactions among Cdk1, Greatwall, and PP2A that function as a second mitotic switch (61, 66, 71), and that avoided the Hill functions used in the two-ODE model. Moreover, the feedback loops between Cdk1 and Wee1 and Cdc25 were omitted, which is a suitable approximation for the situation during cycles 2–12 in *Xenopus* embryos (61, 70, 72). Although this model included a bistable switch and a negative feedback loop, the details of the model are otherwise substantially different from the two-ODE model. Nevertheless, this model could be parameterized to yield realistic cell cycle oscillations (73) (Fig. S6B, Supplementary Note C). Given the computational complexity of scanning over the ten reaction rates present in this model, we exclusively utilized the ABC algorithm. Satisfactory fits were achieved using this model (Fig. S6C), and we observed that pairs of activation energies with high correlations corresponded to antagonistic rates (see Supplementary Note C). The results again show that parameter sets yielding good fits exhibit roughly equal activation energies for faster reactions, and suggest that thermal limits arise from imbalances between cyclin synthesis and degradation rates’ apparent activation energies, echoing our findings in the two-ODE model.

In summary, computational modeling revealed that thermal limits and non-Arrhenius scaling like those seen in early embryos can arise from (at least) two different mechanisms. Firstly, in cases where all rates follow Arrhenius-like scaling but possess varying activation energies, an imbalance emerges, culminating in a thermal limit and a bowed Arrhenius plot. We can call this behavior ‘emergent’, since the limit and the bowing are not inherent to any individual reaction but arise collectively. Secondly, thermal limits can arise if one or more underlying reactions exhibit a thermal optimum and deviate from Arrhenius scaling. Here, the system’s behavior is predominantly dictated by the dynamics of the particular biphasic component(s).

### The durations of interphase and M-phase scale differently with temperature.

To test whether the emergent imbalance model (Fig. 3C, Case 3) contributes to the temperature scaling of the *Xenopus laevis* embryo, we set out to determine how the durations of interphase and M-phase individually scaled with temperature. Both phases contribute to the overall duration of the cell cycle, and the durations of the two phases are largely determined by different process, cyclin synthesis for the former and cyclin destruction for the latter. Because of the opacity of the *Xenopus* embryo, it is difficult to assess these cell cycle phases by microscopy in vivo. We therefore turned to cycling *Xenopus* egg extracts, which are transparent and highly amenable to microscopy.

**Fig. 4:**
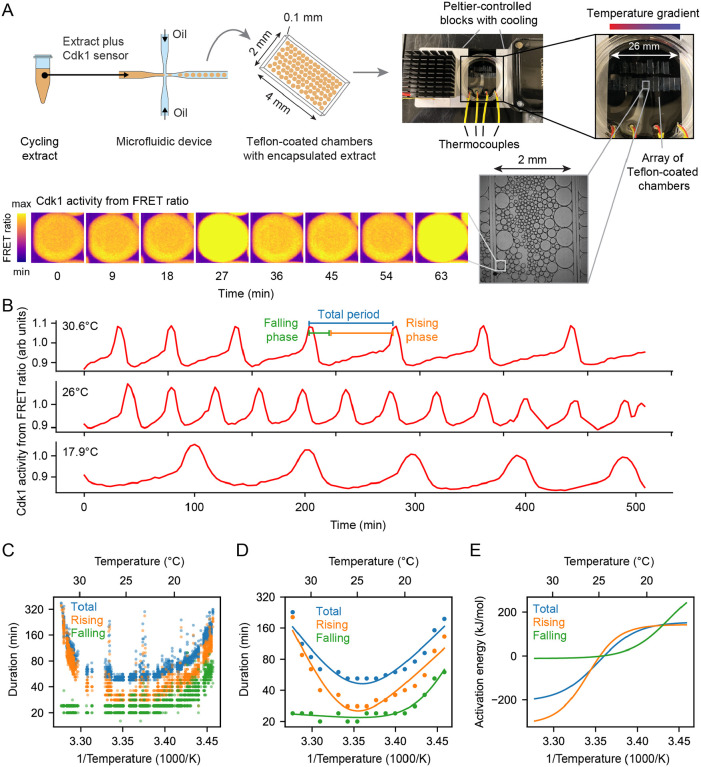
Non-Arrhenius scaling in cycling frog egg extracts. A. Sketch of the setup to encapsulate cycling frog egg extracts in droplets surrounded by oil, including pictures of the customized device to control temperature of extract droplets with snapshots of measured FRET ratios in an example droplet. B. Representative time series of measured FRET ratios at different temperatures. C. Analysis of the duration of the total cell cycle (blue), the rising phase (orange), and the falling phase (green) in function of temperature. D. Mean duration of the data shown in panel E (total period, rising period, falling period) in function of temperature. Optimal fits using a double exponential function are overlayed. E. Local activation energy in function of temperature, calculated from the fitted double exponential function.

Cycling extracts were prepared and supplemented with a Cdk1 FRET sensor whose emission increases when Cdk1 becomes activated and/or its opposing phosphatase(s) is/are inactivated (74), and encapsulated the extract in oil droplets (Fig. 4A), as described (74, 75). The encapsulated extract droplets were then loaded into Teflon-coated imaging chambers, which were immersed in mineral oil and placed on a microscope stage equipped with a custom Peltier element-based heating/cooling device, akin to a previous setup (44). The FRET sensor enabled real-time visualization of oscillations in Cdk1 activity in hundreds of droplets situated at different positions within the temperature gradient (Fig. 4A, Supplemental Video 6). As shown in Fig. 3C (top), Cdk1 activity first rose slowly (phase 1), then spiked to high levels (phase 2), then fell to low levels to allow a new cycle to begin (phase 3). The three phases of the Cdk1 activity cycle correspond well to the phases seen by direct biochemical assays of Cdk1 activities in cycling extracts (66, 76, 77), and to the phases of Cdk1 activation and inactivation seen in the computational models (Fig. 3C, bottom). The cell cycle was found to proceed most rapidly at temperatures of around 25°C, and to slow down at both colder and warmer temperatures (Fig. 4B–C). One unanticipated finding was that the cell cycle proceeded fairly normally at temperatures as high as 32°C, even though in intact embryos, temperatures above 28°C typically killed the embryos and halted the cell cycle. This allowed us to probe a broader range of temperatures in extracts than was possible in vivo. The period of the extracts’ cell cycles increased over time, consistent with previous findings (39, 74, 75, 78). We therefore confined our analysis to the first three cycles, characterizing the duration of each cycle in individual droplets (Fig. S7A–C) and after pooling (Fig. 4C–D). Similar trends could be seen in both the individual and pooled data (Fig. 4C–D). Alternatively, we analyzed all of the cell cycles that occurred during the first 900 minutes rather than the first three cycles. This procedure yield essentially identical results (Fig. S7D).

We analyzed time series data from hundreds of droplets at temperatures from 16°C to 32°C, and plotted the temperature dependence of the cell cycle durations as well as the durations of the rising and falling phases, which correspond approximately to interphase-through-metaphase and metaphase-through-mitotic exit, respectively. This analysis showed that both the total cell cycle duration and the duration of the rising phase exhibited a U-shaped dependence upon temperature—these durations decreased steeply as the temperature rose from 16°C to 20°C, then plateaued, and then increased steeply at temperatures above 30°C (Fig. 4C–D). In contrast, the duration of the falling phase decreased with temperature and then plateaued beginning at about 20°C, but did not slow down to a measurable extent at higher temperatures. These trends can be seen both from the raw data (Fig. 4C) and from binned, averaged data (Fig. 4D). Thus, the rising phase, whose duration is mainly due to the rate of cyclin synthesis, and the falling phase, whose duration is mainly due to APC/C activity, are differently affected by temperature.

Double exponential curves, which assume a biphasic dependence of enzyme activity upon temperature, accounted for the shapes of the Arrhenius plots (Fig. 4D). We computed apparent local activation energies from the fitted curves, which revealed significant changes with temperature (Fig. 4E). At every temperature, the apparent activation energy for total cell cycle duration was close to that of rising phase duration (Fig. 4E), underscoring the fact that interphase constitutes a majority of the cell cycle (Fig. 4B).

### The non-Arrhenius scaling results from both the biphasic temperature sensitivity of cyclin synthesis and an imbalance in the Arrhenius constants for cyclin synthesis and degradation.

**Fig. 5:**
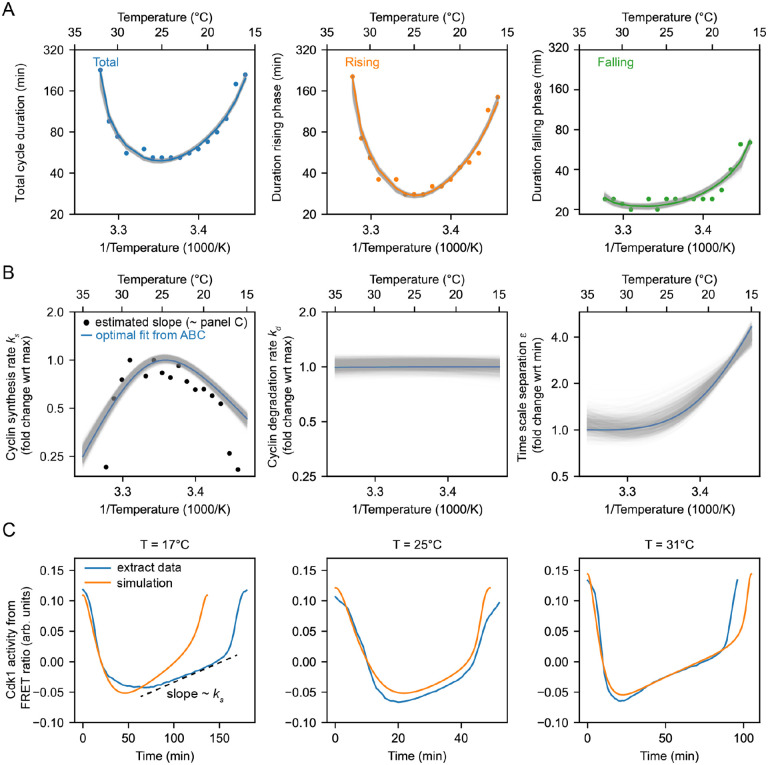
Frog egg extract measurements reveal temperature dependence of cell cycle regulators. A. Using the ABC algorithm, we minimize the mean square error (MSE) between the measured and simulated (using the two-ODE model) durations of rising phase and falling phase. The measurements are shown with the dots, while the gray lines show results from the model with the orange line the best fit (smallest MSE). B. Selected optimal model parameters, i.e. the cyclin synthesis rate, the cyclin degradation rate, and the time scale separation, resulting from the ABC algorithm as shown in A. The black dots correspond to the cyclin synthesis rate $k_{s}$ (nM/min) extracted from the averaged time series as indicated in panel C. C. Blue line: averaged time series of Cdk1 activity (measured FRET ratio) at three different temperatures $\left(T=17^{\circ }C,T=25^{\circ }C,T=35^{\circ }C\right)$. Orange line: time series of the computational model with fitted parameters (best fit - corresponding to the orange lines in panels A-B).

We next asked how well the 2-ODE computational model could account for how the Cdk1 activity cycle varied with temperature in cycling extracts, and whether the scaling of the activation energies for key regulatory processes $\left(k_{s},k_{d},\epsilon \right)$ could be inferred. We utilized the temperature dependence of the measured durations of the rising and falling phases of the Cdk1 time series (Fig. 4D) to optimize our computational model. Employing an exhaustive parameter scan with the approximate Bayesian computation method (details in supplemental note C and Figs. S8–S9), we sought the optimal values for cyclin synthesis rate $\left(k_{s}\right)$, cyclin degradation rate $\left(k_{d}\right)$, and time scale separation (ϵ), which relates to Cdk1 activation (a) and inactivation (i). Leveraging sequential Monte Carlo sampling, the method gradually improved fits to the data, displaying the 200 best fits in gray in Fig. 5A, with the optimal fit highlighted in color. The temperature dependence of the fitted model parameters (Fig. 5B) revealed significant temperature-induced changes in cyclin synthesis rate $\left(k_{s}\right)$ and time scale separation (ϵ), up to five-fold across the temperature range, whereas the cyclin degradation rate $\left(k_{d}\right)$ remained almost constant (see Fig. S8). Additionally, the temperature dependence of $k_{d}$ was well approximated by the (single) Arrhenius equation, whereas $k_{s}$ and ϵ required a double exponential function for accurate description.

Next, we computed the average time series for each cell cycle at various temperatures (blue lines in Fig. 5C, Fig. S10, Supplementary Note 5). These averaged trajectories allowed us not only to ascertain the lengths of interphase (low Cdk1 activity) and the mitotic phase (high Cdk1 activity) but also to determine the duration of transitions between these states (upward and downward switches). Fig. 5A demonstrates that both interphase and M phase durations increased at both high and low temperatures. Intriguingly, while the durations of transitioning into and out of M phase increased at low temperatures, the duration of mitotic exit remained constant at high temperature. Conversely, at high temperatures, the duration of mitotic entry substantially increased. Additionally, we used these average time series to obtain estimates of $k_{s}$ by fitting the slope of interphase time series (Fig. 5C, dashed line, supplemental note C), which revealed similar scaling as obtained by the model fitting with the ABC algorithm (Fig. 5B - overlaid black dots). Implementing the optimized temperature response curves of the model parameters (Fig. 5B) into our computational model, we then analyzed the resulting time series of the cell cycle oscillations (Fig. 5C, orange). The model’s predictions closely aligned with experimental measurements, demonstrating a robust match.

Comparison of apparent activation energies highlights the greater temperature sensitivity of cyclin synthesis rate compared to cyclin degradation rate. This sensitivity aligns with scenarios predicted to yield non-Arrhenius scaling across a wide temperature range (Fig. 3C, Case 3). Furthermore, experimental findings indicate that cyclin synthesis rates decreased at elevated temperatures, corroborating another scenario leading to non-Arrhenius scaling (Fig. 3C, Case 4). Our analysis indicates that both mechanisms contribute to the non-Arrhenius scaling properties of the early embryonic cell cycle oscillator.

### In vitro enzyme assays confirm the imbalance in the cyclin synthesis and degradation $E_{a}$ values.

**Fig. 6:**
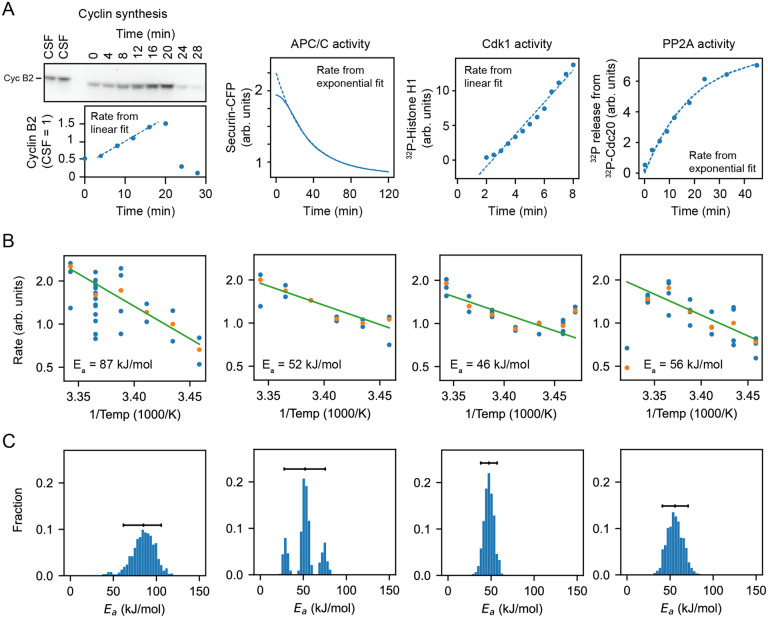
Frog egg extract measurements reveal temperature dependence of cell cycle regulators A. Examples for how the rates for Cyclin B synthesis, APC/C activity, CDK1 activity, and PP2A activity were fitted from time series of different biochemical assays using frog egg extracts at constant temperatures (here for $T=24^{\circ }C$), see Supplementary Note 4. B. The assays were repeated for temperatures in the interval 16–26°*C*, and (apparent) activation energies were extracted. Blue dots represent data of individual fitted time series, while the orange dots are the medians. C. Probability distribution of fitted (apparent) activation energies using bootstrapping see Supplementary Note 2.

To further test the inference that cyclin synthesis and degradation scaled differently at the low end of the temperature range, we carried out direct measurements of the two processes in *X. laevis* frog egg extracts (79) (Fig. 6A–B). The synthesis of one mitotic cyclin, cyclin B2, in cycling extracts was monitored by quantitative Western blotting, using the cyclin B2 levels present in CSF extracts as a normalization standard. APC/C activity was gauged by introducing securin-CFP, translated in wheat germ extracts, as a fluorescent reporter of APC/C activity into CSF extracts, then driving the extracts out of CSF arrest with calcium plus cycloheximide and into mitotic arrest with non-degradable cyclin B. Experimental protocols are detailed in Supplementary Note 4. These measurements were conducted across temperatures ranging from 16°C to 26°C. As shown in Fig. 6, the rate data were consistent the Arrhenius equation (Fig. 6B,green line) and cyclin synthesis was more sensitive to temperature than cyclin degradation, with fitted apparent Arrhenius energies of 87 and 52 kJ/mol, respectively. Bootstrapping supported the statistical significance of this difference (Fig. 6C). This provides direct support for the hypothesis that the different scaling of opposing enzymes contributes to the non-Arrhenius character of the cell cycle period.

We also measured the temperature dependence of two other key cell cycle regulators, cyclin B-Cdk1 and PP2A-B55. These opposing enzymes are critical for the phosphorylation and dephosphorylation of many cell cycle proteins, and their activities would be expected to contribute to the dynamics of mitotic entry and mitotic exit. As shown in Fig. 6, they varied from each other in their temperature sensitivity, with apparent Arrhenius energies of 46 and 57 kJ/mol. Thus, the Arrhenius energies for the pairs of opposing enzymes varied from each other by 1.7-fold and 1.2-fold; evidently this degree of mis-scaling is compatible with robust functioning of the cell cycle oscillator over its nominal temperature range.

### Decreasing the cyclin synthesis rate decreases the viable temperature range.

This analysis suggests that at the high temperature end of the operating range, the *Xenopus* embryonic cell cycle oscillator fails because of the biphasic nature of cyclin B synthesis: the synthesis rate falls as the temperature increases and becomes too low to permit oscillations in the face of the basal levels of APC/C activity. At the low end of the temperature range, the oscillator fails because the difference between the (higher) synthesis and (lower) destruction Arrhenius energies makes the two opposing activities come out of balance, with the synthesis rate again being too low to permit oscillations. If so, the operating range of the cell cycle oscillator is determined by the temperature scaling and balance of two opposing processes particularly critical to the function of the whole circuit.

**Fig. 7:**
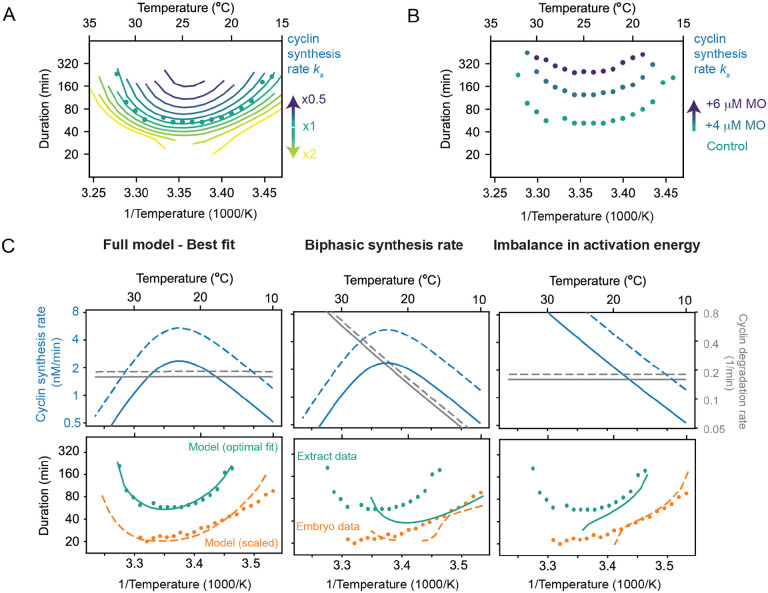
Decreasing the cyclin synthesis rate decreases the viable temperature range A. Influence of the basal cyclin synthesis rate on the shape of the temperature response curves. B. Cell cycle duration in function of temperature obtained from encapsulated extracts with 0, 4, or 6 /*muM* cyclin B morpholino (MO) oligonucleotide. C. Different scenarios in temperature dependence of cyclin synthesis and degradation lead to different non-Arrhenius scaling of cell cycle oscillations. Only the full model, incorporating a biphasic cyclin synthesis rate and an imbalance in activation energies, reproduces the experimental data measured in frog egg extract and in early embryos.

This suggests that concentrations of cyclin B morpholino oligonucleotides that slow but do not halt the cell cycle at normal temperatures should also narrow the temperature range over which the oscillator can operating, decreasing the maximum permissible temperature and increasing the minimum permissible temperature. We tested this idea in the computational model of the oscillator. We extracted the temperature scaling parameters from the data in Fig. 5B, assuming biphasic temperature dependence for the cyclin synthesis rate and (unbalanced) Arrhenius temperature dependencies for the other rates, and then plotted the modeled cell cycle period as a function of the assumed basal cyclin synthesis rate (i.e., the synthesis rate at a nominal reference temperature, which is determined by the magnitude of the pre-exponential coefficient A in the Arrhenius equation). The result is a family of nested U-shaped curves, with both the upper and lower temperature limits of the oscillations moving inward as the assumed cyclin synthesis rate decreases (Fig. 7A). Fig. 7B shows the experimental results for encapsulated extracts in the temperature gradient apparatus with 0, 4, or 6μM cyclin B morpholino oligonucleotide. With increasing morpholino concentration, the minimal period of the cell cycle increases, and both the upper and lower limits of the operating range move inward (Fig. 7B). This indicates that, as hypothesized, the rate of cyclin synthesis (and its balance with cyclin destruction) determines both the operating range of the early embryonic cell cycle oscillator.

Next, we wondered if the model, parameterized based on the data from the *X. laevis* cycling extract, could also reproduce the measured temperature response curve of the cell cleavage rate in early *X. laevis* embryos (Fig. 7C, orange dots). However, the cell cycle oscillations were faster in embryos than in the cycling extracts Fig. 7C, orange vs. green dots). This can be attributed to the dilution of the cytoplasm during the preparation of the cycling extracts, motivated by the observation that a moderate dilution of the extract leads to an increase in the number of cell cycles (80). Therefore, we decided to scale the cyclin synthesis rate and degradation rate such that the cycle duration in extract and embryos matched around 25°C. Strikingly, although we only scaled the rates at one temperature, the whole temperature response curve changed shape capturing the embryo data reasonably well (Fig. 7C, bottom left).

Finally, we asked ourselves if one of the two concurrent mechanisms (biphasic synthesis vs. imbalance of the activation energies) is dominant, and can by itself explain the cell cycle rate in cycling extracts and embryos. Using our model with the optimal parameters, we isolated both effects and observed that they both lead to non-Arrhenius scaling (Fig. 7C, middle-right). However, in isolation, they could not reproduce the experimentally measured response curves, arguing that both mechanisms work together to ensure that the cell cycle oscillator can operate over a wide range of temperatures. Altogether, these findings strongly support our model, which attributes the non-Arrhenius scaling properties of the early embryonic cell cycle oscillator to two concurrent mechanisms. At low temperatures, the scaling is primarily driven by an imbalance between opposing cyclin synthesis and degradation rates. In contrast, at high temperatures, the biphasic nature of cyclin synthesis plays a critical role in capturing the upward curvature of the oscillation period.

## Discussion

Previous work suggested that the early embryonic cell cycle scales similarly with temperature in several organisms (30–33). Here we have extended these measurements to *Xenopus tropicalis* and *Danio rerio*, and have supplemented previous work on *Xenopus laevis* with additional types of measurements. We found that although the periods of the cell cycle at the organisms’ nominal temperatures vary from about 5 min for *C. elegans* and *C. briggsae* to about 25 min for *X. laevis*, the temperature scaling of the periods is quite similar. The apparent Arrhenius energies averaged 76 ± 9 kJ/mol (mean ± S.D., n = 6), and the average $Q_{10}$ value was 2.8 ± 0.4 (mean ± S.D., n = 6) (Figs. 1, 2). In all cases the periods deviated from the Arrhenius relationship at high temperatures, and for *X. laevis*, the Arrhenius plots were non-linear throughout the range of permissible temperatures.

In some ways it is perhaps not surprising that the temperature scaling data could be approximated reasonably well by the Arrhenius equation. Crapse et al. (32) have shown computationally that chaining together a sequence of chemical reactions results in only minor deviations from ideal Arrhenius scaling if one assumes that the individual enzymes’ activation energies do not differ greatly. Experiments have shown that Min protein oscillations, crucial for bacterial cell division, also display Arrhenius-like scaling behaviors (81). The classic chemical oscillator, the Belousov-Zhabotinsky reaction, approximately obeys the Arrhenius equation (82–84), and in general, many biological processes at least approximately conform to the Arrhenius equation or one of the proposed modified versions of the Arrhenius equation (15–22).

These observations notwithstanding, it was not obvious to us why a complex oscillator circuit, with non-linearities and feedback loops, should yield even approximately Arrhenius temperature scaling, and what the origins of the experimentally-observed deviations from Arrhenius scaling might be. Through modeling studies we identified two plausible mechanisms for the observed non-Arrhenius behavior: an emergent mechanism resulting from differences in the Arrhenius energies of opposing enzymes in the network (Fig. 3D, Case 3), and a biphasic temperature dependence for one or more of the critical individual enzymes (Fig. 3D, Case 4). A priori, either or both of these mechanisms could pertain.

Experimental studies on cycling *Xenopus* egg extracts showed that one key step in the oscillator circuit, the synthesis of the mitotic cyclin protein, does in fact exhibit a strongly biphasic dependence on temperature. This was made clear by the fortuitous finding that although intact embryos die above 29°C, cell-free cycling extracts continue to cycle until the temperature exceeds at least 32°C. Above 30°C, the rate of cyclin synthesis and the rate of progression through interphase clearly decrease with increasing temperature, whereas at lower temperatures they increase with increasing temperature (Fig. 4). Our hypothesis is that above some maximum permissible temperature, the imbalance between the cyclin synthesis and degradation rates causes the oscillator to fail and the cell cycle to arrest.

Cyclin synthesis and degradation also scaled differently with temperature at the low end of the permissible temperature range. This was inferred from fitting the parameters of the two-ODE model to the experimental data (Fig. 5), and then was directly shown by in vitro assays of cyclin synthesis and degradation (Fig. 6). This means that below a critical temperature, cyclin synthesis and degradation should again be out of balance, causing oscillations to cease.

To further test this hypothesis, extracts were treated with a morpholino oligonucelotide to inhibit cyclin translation enough to slow but not block the cell cycle at normal temperatures, and asked whether this decreased the maximum permissible temperature, raised the minimum permissible temperature, or both. We found that both temperature limits were similarly affected, and the operating range of the cell cycle oscillator was narrowed, as predicted by our simple two ODE model (Fig. 7). This finding is consistent with the hypothesis that both operating limits are determined by the balance between cyclin synthesis and degradation.

One question then is why did evolution not arrive at a system where cyclin synthesis and degradation did not go out of balance, at high and low temperatures? We suspect that there are trade-offs between competing performance goals for the oscillator and its components. Perhaps the molecular flexibility required to make protein synthesis run as fast as possible at the temperatures typically experienced by an ectotherm render the ribosomes vulnerable to unfolding at slightly higher temperatures. Likewise, perhaps the most efficient cyclin synthesis and degradation activities at normal temperatures happen not to scale with a Arrhenius energies of 69 kJ/mol, as the *X. laevis* oscillator collectively does, and so the observed $E_{a}$ values are a compromise between the highest activities and the ideal scaling behavior.

One final question is how the behaviors seen here compare to those of the same circuit in endotherms, organisms that have at great metabolic cost freed their biochemistry from needing to function reliably over such wide temperature ranges. Although the four enzymatic processes individually assessed here (cyclin synthesis, cyclin degradation, Cdk1 activity, and PP2A-B55 activity) differed in their temperature scaling, they did not differ by that much; their $E_{a}$ values averaged to 72 kJ/mol with a standard deviation of 22 kJ/mol or 30%. It seems plausible that endothermy might allow enzymes with a wider range of activation energies to be used than would be possible in ectotherms.

## Materials and Methods

### Xenopus egg extract.

Cell-free cycling extracts and CSF extracts were made from *Xenopus laevis* eggs following a published protocol from Murray (85). For cycling extracts, this protocol was adapted as in (75). Extracts for Fig. 3B–G and Fig. 4 were then supplemented with 1μM Cdk1-FRET sensor, as described in Maryu and Yang (74), and also with 1X energy mix (7.5 mM Creatine phosphate, 5mM ATP, 1mM EGTA, 10 mM MgCl2). Work from the Yang lab demonstrated that an intermediate range of dilution of the extracts can improve the number of cycles, with the best activity at around 30% dilution (80). As a result, for the data described here, the dilution was kept constant at 30% with extract buffer (100 mM KCl, 0.1 mM CaCl2, 1 mM MgCl2, 10 mM potassium HEPES, 50 nM sucrose, pH 7.8). Extracts for the biochemical assays in Fig. 3A were undiluted.

The extract was encapsulated via a water-in-oil emulsion using a micrufluidic device. The fabrication of the device and droplet generation followed a previously published protocol (86). Briefly, cycling extract (water phase) was mixed with 2% 008-FluoroSurfactant in HFE7500 (Ran Biotechnologies, Inc.) (oil phase) inside a microfluidic device driven by an Elveflow OB1 multi-channel flow controller (Elveflow). Air pressure was 2 psi for both the extract and oil channels. After droplets were generated, they were loaded into VitroCom hollow glass tubes with a height of 100μm (VitroCom, 5012) pre-coated with trichloro (1H, 1H, 2H, 2H-perfluorooctyl) silane, and then immersed into a glass-bottom dish (WillCo Wells) filled with heavy mineral oil (Macron Fine Chemicals) to prevent evaporation.

### Temperature gradient generation.

A custom plastic microscope stage was fabricated to fit two aluminum plates on each side of the imaging dish. Each aluminum plate was attached to a TEC1-12706 40*40MM 12V 60W Heatsink Thermoelectric Cooler Cooling Peltier Plate (HiLetGo) using thermal conductive glue (G109, GENNEL). The plate designated for temperatures above room temperature had an additional heatsink (40mm × 40mm × 20mm, black aluminum, B07ZNX839V, Easycargo) and a cooling fan to improve performance. The plate designated for cold temperatures had an additional liquid cooling system (Hydro Series 120mm, CORSAIR) attached with termal conductive glue.

Peltier devices were controlled via two CN79000 1/32 DIN dual zone temperature controllers (Omega). In all experiments, the target temperature was set to 65°C and 1°C for the hot and cold plates respectively. With both plates on, it was always the case that the hot plate reached its target temperature and stayed constant within 5–10 min and the cold plate stayed stable at 10°C.

The imaging dish was attached to the aluminum plates with Thermal adhesive tape 2-5-8810 (DigiKey) to ensure proper thermal conduction. Temperature was logged via 4 K-Bead-Type thermocouples placed on the imaging dish touching the bottom surface. Data was acquired using a 4 Channel SD Card Logger 88598 AZ EB (AZ Instruments). Room temperature was also captured using the same method via a thermocouple attached to the microscope stage.

### Western blotting.

Cycling extracts were prepared according to the method by Murray et al. (85), except that eggs were activated with calcium ionophore A23187 (5μl of a 10 mg/ml stock of A23187 in 100 ml 0.2x MMR) rather than with electric shock. After preparing the extracts, they were distributed to several eppendorf tubes and brought to a specified temperature between 16 and 26°C within 20 minutes. 2μl samples were taken every 4 minutes (every 8 minutes at 16°C) and immediately frozen on dry ice. To each 2μl aliquot, 48μl of SDS sample buffer supplemented with DTT was added, and the samples were boiled during 10 minutes at 95°C. 12μl of the cycling extract samples and 4μl of the reference samples (CSF extract prepared according to the method by Murray et al.(85), were run on 10% Criterion TGX Precast protein gels and transferred to a PVDF membrane using the Bio-Rad Trans-blot Turbo system. After blocking in milk (4% w/v in TBST), the blots were incubated with a 1/500 dilution of anti-cyclin B2 antibody (X121.10, Santa Cruz) overnight at 4°C followed by a 1/10.000 dilution of anti-mouse IgG HRP-linked whole secondary antibody (GE Healthcare NA931), during 1 hour at room temperature. Finally, the blots were developed using Supersignal West Femto chemiluminescent substrate.

### Time-lapse fluorescence microscopy.

For Figs. 4, 5, 7, imaging was carried out on an inverted Olympus IX83 fluorescence microscope with a 4× air objective, a light emitting diode fluorescence light source, a motorized x-y stage, and a digital complementary metal–oxide–semiconductor camera (C13440–20CU, Hamamatsu). The open-source software μManager v1.4.23 was used to control the automated imaging acquisition. Bright-field and multiple fluorescence images of CFP, FRET, and YFP were recorded at a frequency of one cycle every 3 to 7 min for 40 to 50 hours for each sample.

### Image processing and analysis methods.

Grids of images were captured and subsequently stitched together using ImageJ’s Grid/Pairwise Stitching plug-in, in conjunction with additional pipeline code written in Fiji/Java. Bright-field images from the first frame were used to generate stitching parameters, which were fed to ImageJ to stitch each channel at each frame consecutively. The FRET ratio was calculated as in Maryu and Yang (74).

For Figs. 4, 5, 7, custom scripts in MATLAB 2020a and ImageJ were written to perform image processing. Briefly, each microscope position was processed by manually selecting the region containing the tube of interest and then algorithmically cropping and resizing that region in all channels. Then, bright-field images were used for individual droplet segmentation and tracking using Trackmate 7.12.1. Only individual droplets whose radius was smaller than 100 μm and track started within the first 60 min of the experiment were selected for further analysis. FRET ratio intensity peaks and troughs were first auto-selected and then manually checked and corrected using custom Python scripts. Rising and falling periods were calculated from this data. All code is available at https://github.com/YangLab-um/temperature and https://github.com/YangLab-um/dropletDataProcessing.

## Supplementary Material

Supplement 1

## Data Availability

All experimental data have been deposited at [upcoming], and are publicly available as of the date of publication. All original modeling code has been deposited at the Gelens Lab Gitlab [upcoming] , and is publicly available as of the date of publication. All codes for image processing and analysis methods can be found in https://github.com/YangLab-um/temperature and https://github.com/YangLab-um/dropletDataProcessing, publicly available as of the date of publication. Any additional information required to reanalyze the data reported in this paper is available from the lead contact upon request.
