# Supplementary material for: Mechanistic origins of temperature scaling in the early embryonic cell cycle
Source: bioRxiv. 2025 Feb 4:2024.12.24.630245. Originally published 2024 Dec 24. Preprint. [Version 2] doi: 10.1101/2024.12.24.630245 (PMC11703202; doi:10.1101/2024.12.24.630245)
Supplement: Supplementary file 1 [file media-1.pdf]

Supplementary Note 1: Determination of local E_a and Q_{10}

In the case of a duration or rate that scales according to the Arrhenius equation

$$k = Ae^{\frac{-E_a}{RT}} \tag{13}$$

the activation energy is a constant. The formula above is equivalent to

$$\ln k = \ln A - \frac{E_a}{R}\frac{1}{T}. \tag{14}$$

The value of E_a can thus be calculated from the slope of the line obtained when plotting $\ln k$ vs $1/T$. Or,

$$E_a = -R \times \frac{d(\ln k)}{d(1/T)}. \tag{15}$$

This equation can also be used as the definition of a local activation energy $E_a(T)$ for any function $k(T)$.

Similarly, we can define a local Q_{10} value. In this section we explain how a Q_{10} value can be obtained for a process with temperature-dependent rate. The Q_{10} is the fold change in rate when the temperature increases by 10 degrees Celsius. If the value of Q_{10} is constant over all temperatures, the rate dependence should have the form

$$k \sim Q_{10}^{T/10}.$$

This form is different from the Arrhenius equation. It would therefore be incorrect to say of a process that its activation energy E_a *and* its Q_{10} are both constant over a range of temperatures.

The Q_{10} can be calculated as

$$Q_{10} = \left(\frac{k(T_2)}{k(T_1)}\right)^{\dfrac{10}{T_2 - T_1}},$$

where $k(T_i)$ is the rate of the process calculated at temperature T_i. Since this formula holds for any choice of T_1 and T_2, we can look at the limit $T_2 \to T_1$ and use this formula to define a *local* Q_{10}, $Q_{10}(T)$. The use of 'local' for a number that is meant to convey what happens over a temperature change of 10 degrees is a bit contradictory, but we will make abstraction of this and use $Q_{10}(T)$ to indicate a local sensitivity to temperature.

If the rate of a process depends on temperature through any (differentiable) function $k(T)$, then for any h we would have

$$Q_{10}(T) = \left(\frac{k(T+h)}{k(T)}\right)^{\frac{10}{h}}$$

A Taylor expansion for small h gives that this is approximately equal to

$$\left(1 + h\frac{k'(T)}{k(T)}\right)^{\frac{10}{h}},$$

where $k' = \frac{dk}{dT}$. Using the definition of the exponential function, this goes to

$$\exp\left(10\frac{k'(T)}{k(T)}\right)$$

for small h. We thus define the local Q_{10} as

$$Q_{10}(T) = e^{10\frac{k'(T)}{k(T)}}. \tag{16}$$

We can also express the formula for the local E_a (Eq. (15)) using the derivative of the rate:

$$E_a(T) = -R\frac{d(\ln k)}{d(1/T)} = -R\frac{1}{k}\frac{dk}{d(1/T)} = R\frac{k'}{k}T^2.$$

This also gives a link between local Q_{10} and E_a:

$$Q_{10}(T) = \exp\left(10\frac{E_a(T)}{RT^2}\right). \tag{17}$$

Supplementary Note 2: Bootstrapping

The histograms that describe the uncertainty on the fitted activation energies on the rates, shown in Figs. 1 and 6, were obtained using a bootstrapping procedure. First, we generated a new dataset by resampling the rate measurements *with replacement* from the original data. In this case, we used a stratified bootstrap: we resampled per temperature. So if in the original dataset, there were three values of E_a for a given temperature, in a bootstrapped dataset there will also be three values for that temperature, and they are resampled with replacement from the original three. For each bootstrapped dataset, we inferred the activation energy by fitting a straight line in the Arrhenius plot. We fitted on the median per temperature. We did this for 1000 bootstrapped datasets, and these 1000 values of E_a are represented in the histograms.

Supplementary Note 3: Computational cell cycle models

A. Two-ODE cell cycle model. As described in the main text, we made use of a two-ODE cell cycle model based on one originally described in (67):

$$\begin{aligned} \frac{d\text{cyc}}{dt} &= k_s - k_d d[\text{cdk1}_a]\text{cyc}, \\ \epsilon \frac{d\text{cdk1}_a}{dt} &= k_s - k_d d[\text{cdk1}_a]\text{cdk1}_a + k_a a[\text{cdk1}_a](\text{cyc} - \text{cdk1}_a) - k_i i[\text{cdk1}_a]\text{cdk1}_a, \end{aligned} \tag{18}$$

The first equation describes how cyclin B (cyc) is synthesized throughout the cell cycle at a rate k_s (nM/min) and how it is degraded at a rate k_d (1/min) by the proteasome after ubiquitination by active APC/C. APC/C is assumed to be activated instantaneously by active cyclin B - Cdk1 complexes (abbreviated as cdk1_a) in an ultrasensitive way (given by $d[\text{cdk1}_a]$). The second ODE describes the time evolution of active cyclin B - Cdk1 complexes, assuming that all synthesized cyclin B quickly binds to Cdk1 to form a complex. Moreover, the positive feedback of Cdk1 via Cdc25, and the double negative feedback of Cdk1 via Wee1 are included as an activating ($a[\text{cdk1}_a]$) and inhibiting ($i[\text{cdk1}_a]$) ultrasensitive function as well, motivated by direct experimental measurements of those response functions (68, 69). The different ultrasensitive functions have the following form:

$$a[x] = a_{\text{Cdc25}} + b_{\text{Cdc25}} \frac{x^{n_{\text{Cdc25}}}}{\text{EC}_{50,\text{Cdc25}}^{n_{\text{Cdc25}}} + x^{n_{\text{Cdc25}}}}, \tag{19}$$

$$i[x] = a_{\text{Wee1}} + b_{\text{Wee1}} \frac{\text{EC}_{50,\text{Wee1}}^{n_{\text{Wee1}}}}{\text{EC}_{50,\text{Wee1}}^{n_{\text{Wee1}}} + x^{n_{\text{Wee1}}}}, \tag{20}$$

$$d[x] = a_{\text{APC}} + b_{\text{APC}} \frac{x^{n_{\text{APC}}}}{\text{K}_{\text{APC}}^{n_{\text{APC}}} + x^{n_{\text{APC}}}}. \tag{21}$$

This type of model has previously been used to successfully describe various aspects of cell cycle oscillations (34, 67, 70). In the present work, we simplified this model further as follows, to have a clearer separation of the first ODE with cyclin synthesis and degradation, and the second ODE just describing Cdk1 activation and inactivation processes. The results are very similar and do not influence the conclusions in this work.

$$\begin{aligned} \frac{d\text{cyc}}{dt} &= k_s - k_d d[\text{cdk1}_a]\text{cyc}, \\ \epsilon \frac{d\text{cdk1}_a}{dt} &= k_a a[\text{cdk1}_a](\text{cyc} - \text{cdk1}_a) - k_i i[\text{cdk1}_a]\text{cdk1}_a, \end{aligned}$$

Using experimentally motivated parameters (67), model (22) reproduces cell cycle oscillations with a period of approx. 30 min (Fig. 3C). These oscillations manifest as a closed trajectory, a limit cycle, in the (cyc, cdk1_a) phase plane (Fig. 3B, red), helping to better understand their existence via the intersection of nullclines (NCs). NCs are defined by points where $\frac{d\text{cyc}}{dt} = 0$ (Cyc NC) or $\frac{d\text{cdk1}_a}{dt} = 0$ (Cdk1 NC). When $\epsilon << 1$, oscillations occur at the intersection of the cyclin NC and the middle branch of the S-shaped Cdk1 NC (as depicted in Fig. 3B).

Symbol	Meaning	Value
k_s	Cyclin production rate	1.25 nM/min
k_d	Cyclin degradation rate	$0.1\ \mathrm{min}^{-1}$
k_i	Maximal Wee1 activity	$1\ \mathrm{min}^{-1}$
k_a	Maximal Cdc25 activity	$1\ \mathrm{min}^{-1}$
a_{Cdc25}	Basal Cdc25 activity	0.2
b_{Cdc25}	Maximal increase in Cdc25 activity	0.8
K_{Cdc25}	Threshold for Cdc25 activation	30 nM
n_{Cdc25}	Hill exponent for Cdc25 activation	10
a_{Wee1}	Basal Wee1 activity	0.1
b_{Wee1}	Maximal increase in Wee1 activity	0.4
K_{Wee1}	Threshold for Wee1 activation	30 nM
n_{Wee1}	Hill exponent for Wee1 activation	5
a_{APC}	Basal APC/C activity	0.1
b_{APC}	Maximal increase in APC/C activity	0.9
n_{APC}	Hill exponent for APC/C activation	15
K_{APC}	Threshold for APC/C activation	30 nM
ϵ	Timescale parameter	0.1

Table 1: Parameter values used in the 2-ODE cell cycle model. These are the basal values that correspond roughly to the period of the *X. laevis* and *D. rerio* cell cycle. To describe the *X. tropicalis cycle*, which is faster, we multiply k_s and k_d by 1.3. The basal values of k_s, k_d and ϵ are also different for extracts: these values are obtained as part of the fitting to the time series.

B. Five-ODE mass action model.

B.1. The model. We used a five-equation model for relaxation oscillations arising out of the interaction between Cdk1, Greatwall and PP2A (Fig. S5A). This pathway is a different part of the mitotic control system, which underlies the second mitotic switch. In the two-ODE model used in the main text, Cdk1 is involved in two feedback loops, through Wee1 and Cdc25. These feedbacks lead to bistability of Cdk1 activity as function of cyclin B levels. In the five-equation model, these feedback loops are not present, as is appropriate for cycles 2–12 in embryos (70). Here, any cyclin B-Cdk1 complex is directly activated assuming Cdk1 is present in excess over cyclin B: the response curve of Cdk1 activity as function of cyclin B levels would be linear. The production rate of cyclin B therefore directly corresponds to the production rate of active cyclin B-Cdk1 complexes. The equations we use are based on the paper by Hopkins et al. (65), who show that the equations for Greatwall, ENSA and PP2A lead to bistability. We complement their system with equations for Cdk1 and APC/C to turn the bistable system into a relaxation oscillator (75) (Fig. S5B).

We use the following equations:

$$u' = k_{p,a}(A_T - u)v - k_{d,a}u(P_T - y) \quad \mathbf{(22)}$$
$$v' = k_s - k_d uv \quad \mathbf{(23)}$$
$$w' = k_{p,g}(G_T - w)v - k_{d,g}(P_T - y)w \quad \mathbf{(24)}$$
$$x' = -k_{p,e}xw + k_{\mathrm{cat}}y \quad \mathbf{(25)}$$
$$y' = k_{\mathrm{ass}}(E_T - x - y)(P_T - y) - k_{\mathrm{diss}}y - k_{\mathrm{cat}}y. \quad \mathbf{(26)}$$

Here, u denotes active APC/C, v are the active cyclin B-Cdk1 complexes. The variable w corresponds to phosphorylated Greatwall, x is free, unphosphorylated ENSA and y is the complex ENSA-PP2A. The parameters of the model are the biochemical rates and the total amounts of APC/C (A_T), Greatwall (G_T), ENSA (E_T), and PP2A (P_T).

The first equation describes the activity of APC/C. We assume that APC/C can be converted from its inactive (unphosphorylated) form to its active (phosphorylated) form by Cdk1 through mass-action kinetics. The dephosphorylation is performed by PP2A. Note that y is the concentration of the ENSA-PP2A complex and P_T is total PP2A, such that the available PP2A is $P_T - y$. The second equation describes cyclin B-Cdk1 levels. These are governed by production and degradation of cyclin B, the latter of which is modeled through mass action. The equation for active Greatwall (variable w) describes the conversion between active and inactive Greatwall by Cdk1 and PP2A respectively — analogous to the APC/C equation. The fourth equation describes free unphosphorylated ENSA. This concentration decreases through phosphorylation of ENSA by Greatwall, and it increases through the dephosphorylation, which is mediated by PP2A. The final equation describes the concentration of the ENSA-PP2A complex.

The parameters, their meaning and their standard values can be found in Table 2. The parameter set is not based on experimental values, but was chosen to obtain a relaxation oscillation of amplitude (in the Cdk1 variable) and period that correspond to observations. All variables except for cyclin B-Cdk (v) are in arbitrary units, this is why the units of the rate constants look a bit awkward. As for the 2-ODE model, k_s and k_d are multiplied by 1.3 if simulations are to be compared with data from *X. tropicalis*.

Symbol	Meaning	Value
$k_{p,a}$	phosphorylation rate of APC/C by Cdk1	0.4 $\mathrm{nM}^{-1}\mathrm{min}^{-1}$
$k_{d,a}$	dephosphorylation rate of APC/C by PP2A	100 min^{-1}
$k_{p,g}$	phosphorylation rate of Greatwall by Cdk1	0.06 $\mathrm{nM}^{-1}\mathrm{min}^{-1}$
$k_{d,g}$	dephosphorylation rate of Greatwall by PP2A	20 min^{-1}
$k_{p,e}$	phosphorylation rate of ENSA by Greatwall	6 min^{-1}
k_{ass}	association rate of phosphorylated ENSA and PP2A	100 min^{-1}
k_{diss}	dissociation rate of ENSA-PP2A complex	1 min^{-1}
k_{cat}	rate of catalyzed dephosphorylation of ENSA	4.5 min^{-1}
k_s	Cyclin production rate	1.5 nM/min
k_d	Cyclin degradation rate	0.15 min^{-1}
A_T	Total APC/C in the system	1
G_T	Total Greatwall in the system	1
E_T	Total ENSA in the system	3
P_T	Total PP2A in the system	1

Table 2: Parameter values used in the mass action model.

B.2. Interpretation in the phase plane. The model with only Greatwall, ENSA and PP2A (w, x, y) has been shown to produce a bistable response as function of the amount of active Cdk1 (v) (65). In our version, we added production of cyclin B and its degradation through ubiquitination by APC/C to turn the bistable system into a relaxation oscillator. Even though the system is five-dimensional, we can understand it in the phase plane. To do this, we perform a reduction to a two-variable system. We assume a quasi-steady-state condition on w, x and u, and set their derivatives to zero. When we do this, we assume that these variables evolve on a faster timescale than the others. We do expect that the levels of cyclin B-Cdk1 evolve on a slower timescale than the other variables: production and degradation are slower than the phosphorylation and dephosphorylation reactions. The reasons for keeping y as the additional variable and not one of the others is more practical: taking $w' = x' = u' = 0$ leads to explicit expressions for these variables as function of v and y, which does not work if we take, say, u as remaining variable. We find

$$
\begin{aligned}
w(v,y) &= \frac{1}{1+\frac{k_{d,g}(P_T-y)}{k_{p,g}v}} G_T \\
x(v,y) &= \frac{k_{\text{cat}}y}{k_{p,e}w} = \frac{k_{\text{cat}}y}{k_{p,e}G_T}\left(1+\frac{k_{d,g}(P_T-y)}{k_{p,g}v}\right) \\
u(v,y) &= \frac{1}{1+\frac{k_{d,a}(P_T-y)}{k_{p,a}v}} A_T.
\end{aligned} \tag{27}
$$

Using these, we can reduce the system to two equations:

$$
\begin{aligned}
v' &= k_s - k_d u(v,y) v \\
y' &= k_{\text{ass}}(E_T - x(v,y) - y)(P_T - y) - k_{\text{diss}}y - k_{\text{cat}}y.
\end{aligned} \tag{28}
$$

Fig. S5B shows the phase plane of this system with the associated limit cycle. The projection of the solution of the five-ODE system for the same parameter values is also shown. The two limit cycles are close in the phase plane, but their period is significantly different (Fig. S5B). This reduction shows that we can qualitatively understand the oscillations of this system in the phase plane. In particular, we confirm that the limit cycle is of relaxation type and goes around the underlying bistable switch. The cycle is, as in the 2-ODE model, driven by cyclin B accumulation and degradation. Once cyclin B-Cdk1 levels cross a threshold, the activity of the phosphatase is quickly suppressed. This allows activation of APC/C, which leads to cyclin degradation and brings the system back to a state of low Cdk1 activity. In particular, we can see from Eq. (28) that the production and degradation rates affect the non-S-shaped nullcline only.

For this model, we studied how the period of the oscillation changes if each of the parameters has a dependence on temperature. We analyzed how different activation energies for the different parameters can lead to different scaling as well as thermal

ranges. As for the Yang-Ferrell model, this analysis can be interpreted in the phase plane, by examinining how parameter changes affect the location of the nullclines. From the phase-plane picture we can see that, when the oscillations disappear, the system becomes stuck in a state with either high or low phosphatase activity. As before, which one it is will depend on the relative magnitude of $E_a(k_s)$ and $E_a(k_d)$.

The steady state of the system only depends on the ratios of the following parameters:

$$\frac{k_s}{k_d}, \quad \frac{k_{p,a}}{k_{d,a}}, \quad \frac{k_{p,g}}{k_{d,g}}, \quad \frac{k_{p,e}}{k_{\text{cat}}} \text{ and } \frac{k_{\text{ass}}}{k_{\text{cat}}+k_{\text{diss}}}.$$

It follows that, for any set of activation energies such that

$$\begin{aligned} E_a(k_s) = E_a(k_d), &\quad E_a(k_{p,a}) = E_a(k_{d,a}), \\ E_a(k_{p,g}) = E_a(k_{d,g}), &\quad E_a(k_{p,e}) = E_a(k_{\text{cat}}) = E_a(k_{\text{ass}}) = E_a(k_{\text{diss}}), \end{aligned}$$

the steady state of the system is independent of temperature. Under the assumption that the relative magnitude of the timescales stays the same, this means that we would expect oscillations over a large range of temperatures if these rates scale in a similar way. These ratios usually have the rates for two counteracting reactions in numerator and denominator.

C. Details on the parameter sweep for fitting the 2-ODE model to embryo data. In Fig. 3E, we show fits of the 2-ODE model to the duration of the embryonic cycle in different cases. These were obtained by performing computational parameter sweeps. All nonscaling parameters were as in Table ??, and for *X. tropicalis* we multiplied k_s and k_d by 1.3. We kept k_i and k_a and only scale k_s and k_d.

In particular, we performed two sweeps:

1. Both k_s and k_d scale Arrhenius, but their activation energies differ. We varied $E_a(k_s)$ and $E_a(k_d)$ from 0 to 150 kJ/mol in steps of 5 kJ/mol. For each combination of E_a, we perform simulations for temperatures from 0 to 50°C and save the period to file. Next, we calculate the distance between simulated periods and the duration from the data as follows. First, if the simulation did not yield oscillations for the full range of temperatures in the dataset, we consider the error infinite. In the other case, we calculate the Mean Squared error on the logarithms of the durations.

   The curves shown in Fig. 3E, Case 1 and 3, are obtained by finding the $E_a(k_s)$ and $E_a(k_d)$ that minimize the MSE under the constraint that $E_a(k_s) = E_a(k_d)$ (Case 1, green line), $E_a(k_s) > E_a(k_d)$ (Case 3, orange line) and $E_a(k_s) < E_a(k_d)$ (Case 3, red line).

2. For the biphasic response, we assumed that the temperature scaling of k_s is given by a double-exponential curve

   $$k_s = A_1 e^{-\frac{E_1}{RT}} + A_2 e^{-\frac{E_2}{RT}}.$$

   In this parameter sweep, k_d scales Arrhenius and has the same activation energy as k_s for the lower-temperature regime. The difference now is the second exponential term which leads to a bending of the curve for k_s at high T.

   We performed a sweep over E_1 and E_2, with E_1 ranging between 0 and 150kJ/mol and E_2 between -150 and 0 kJ/mol. We determined the prefactors by fixing the rate of k_s at a reference temperature at 18°C, and by fixing the optimal temperature T_m. We also scanned over different values of T_m around the optimal temperature from the data, in particular we simulated for T_m for integer values of the temperature between $T_{m,\text{data}} - 7$ and $T_{m,\text{data}} + 7$, where $T_{m,\text{data}}$ is 28°C for *X. laevis*, 30°C for *X. tropicalis* and 32°C for *D. rerio*.

   Distance between model simulaton and data was determined as above.

D. Fitting temperature-dependent computational models to data using the ABC algorithm.

D.1. Fitting cycling extract data with the two-ODE model. In Fig.5 we show the results of fitting the parameters to the time scaling of extract data. These fits were obtained using Approximate Bayesian Computation - Sequential Monte Carlo (ABC-SMC) (71). This algorithm sequentially samples parameter sets that provide better and better fits to the data. The output of the algorithm is N parameter sets Θ_i with associated weights w_i. Each of these parameter sets provides a fit closer than a prescribed distance ε to the data. The N weighted parameter sets constitute a sample from the posterior distribution

$P(\Theta \mid d(x^*, x_0) < \varepsilon)$, where x_0 is the data, x^* is the data resulting from a simulation with parameters Θ and d is a distance function. If ε is small, this distribution approximates the posterior distribution $P(\Theta \mid x_0)$: the probability that a parameter set Θ is the true one, given the observed data. In ABC-SMC, the value of ε is lowered over the course of different generations as a way of getting better and better approximations of the posterior. We use the implementation of this algorithm given in pyABC (72).

For the extract fits, we describe the temperature scaling of each of the rates k_s, k_d and ϵ using a double-exponential formula. Instead of the four parameters A_1, E_1, A_2, B_2 that go into a formulation of the form

$$\text{rate} = A_1 e^{\frac{E1}{RT}} + A_2 e^{\frac{E2}{RT}},$$

we decide to use more interpretable parameters: the basal value of the rate at 18 degrees Celsius, the temperature T_m at which is maximal (minimal for ϵ) value is obtained, and the two activation energies E_1 and E_2. Note that we can map k_0, T_m, E_1, E_2 to A_1, E_1, A_2, E_2 directly.

A parameter vector Θ contains 12 values $(E_{1,k_s}, E_{2,k_s}, T_{m,k_s}, k_{s,0}, E_{1,k_d}, E_{2,k_d}, T_{m,k_d}, k_{d,0}, E_{1,\epsilon}, E_{2,\epsilon}, T_{m,\epsilon}, \epsilon_0)$. Each parameter set thus defines three functions $k_s(T)$, $k_d(T)$ and $\epsilon(T)$. We simulate the 2-ODE model over the temperatures from the dataset, using the rates defined by these scaling functions. All the other rates are kept to their basal value (Table **??**). For each temperature, we detect whether the system is oscillating using peak-detection as described in Methods. For oscillating systems, we then use the *cdk* timeseries to determine the rising (min to max) and falling (max to min) durations.

The output of the simulation is thus $x_s = (R_i, F_i)$: the duration of rising and falling part of the cycle, for each temperature T_i in the dataset. These data from the simulation are then compared to the same data obtained from the extract time series x_d. The distance function we use is

$$d(x_d, x_s) = \frac{1}{N} \sum_i |\ln F_{d,i} - \ln F_{s,i}| + |\ln R_{d,i} - \ln R_{s,i}|. \quad \textbf{(29)}$$

We thus take the differences of the rising and falling times, for each temperature, and take the average of the absolute values. Note that we use the logarithms of the durations. The durations can vary quite a bit in absolute value, and we use the logarithms to prevent the algorithm being skewed to approximating the large durations (at extreme temperatures). We take $d(x_d, x_s) = \infty$ if there is a temperature in the dataset for which the simulation did not produce an oscillation. The consequence is that we only search for parameter values for which the model produces oscillations over *at least* the range we see in the experiment.

For the extract simulations, we use 1000 particles per generation. The prior distributions were uniform distributions for all parameters, for the E_1 between 0 and 200 kJ/mol, for the E_2 between -200 and 0 kJ/mol, for the T_m between 10 and 45 degrees Celsius, for $k_{s,0}$ between 0 and 5, for $k_{d,0}$ between 0 and 2 and for ϵ_0 between 0 and 100.

We let the algorithm run with for 40 generations and inspected visually the resulting fits described. We did not retain the last generation for the figures shown, because these fit the data too closely. Since the data itself has variability we did not want to overfit. We picked the 35th generation for the results in the main text. Fig. S9 shows the marginal parameter distributions in this generation. All the parameters for k_s, as well as $k_{d,0}$ and ϵ_0, are clearly centered on one value. Moreover, E_{1,k_d} and E_{2,k_d} are peaked close to zero. For the other parameters, the distribution is much wider. This is also clear from the plots in Fig. S10: These show the marginal distribution of the different parameters over the different generations of the ABC SMC run. For the parameters peaked around one value, we see clear convergence of the posteriors. For the others, this is less clear.

D.2. Fitting embryo data with the two-ODE model. Here we describe the setup of the ABC algorithm for fitting the embryo data using purely Arrhenius scaling on the rates (Fig. S4). We use the two-ODE cell cycle model to capture the period scaling observed in the data for *Xenopus laevis*, *Xenopus tropicalis* and *Danio rerio*.

For this model we use four different parameters: the activation energies of k_s, k_d and of the 'activation' and 'inactivation' reactions. The 'activation' can be thought of as scaling the dephosphorylation rate of Cdk1 by Cdc25. It modifies the parameters a_{Cdc25} and b_{Cdc25} in the model. The backward rate scales phosphorylation by Wee1, corresponding to the parameters a_{Wee1} and b_{Wee1}. Each simulation produces the period P_i of the oscillation for each of the temperatures T_i in the embryo dataset (or zero if for a temperature there is no oscillation).

We compare the distance between the simulated dataset x_s and the observed data x_d (the durations of the embryonic cycles) with the distance function

$$d(x_d, x_s) = \frac{1}{N} \sum_i |\ln P_{d,i} - \ln P_{s,i}|. \quad \textbf{(30)}$$

The distance is set to infinity if the simulation does not produce an oscillation for any of the temperatures T_i.

The basal rates at 18°C are as in Table ??, but for *X. laevis* we multiply k_s and k_d by 1.3 to account for its faster cycle. Since the temperature scaling of a rate is defined by

$$\text{rate} = \text{basal rate} \times e^{\frac{-E_a}{R}\left(\frac{1}{T}-\frac{1}{T_0}\right)},$$

only varying the E_a will lead to always the same value of the rate at T_0, 18 degrees Celsius in our case. This means that all period curves will go through the same point at 18 degrees. To circumvent this, we include an additional parameter which adds an overall scaling of the period, by scaling the basal values of k_s and k_d. In all the samples, this parameter is very close to one.

We use 200 particles in the ABC run and let the algorithm run for 15 generations. The prior distributions for all activation energies were uniform on $[0, 150]$ kJ/mol. Fig. S4 shows the resulting fits from the ABC algorithm.

The two-dimensional probability densities in Fig. S4B are projections of the points ($E_a(k_s)$, $E_a(k_d)$,$E_a(k_a)$, $E_a(k_i)$) into two different planes. Lighter color means higher probability. The bottom row in this figure shows a white cloud along the diagonal. This means that the best fits to the data are obtained when $E_a(k_a)$ ≈E$_a(k_i)$.Theupperheatmapsshowthattheactivationenergiesof k$_s$ and k_d need to be different (off-diagonal clouds) for good fits. Whereas for *X. tropicalis* and *D. rerio*, good fits are obtained when $E_a(k_s)$ is larger than $E_a(k_d)$, for *X. laevis* there are two off-diagonal clouds. These density plots were obtained from the 200 weighted points that are the output of the ABC algorithm, smoothed using a Gaussian kernel. In Fig. S4A we show the actual fits corresponding to each of these sampled points in gray, with more transparent lines corresponding to parameter sets with lower weight. In orange, we show the best fit (lowest distance). The fits are quite good overall, but the upper thermal limit and the bend upwards are not so easy to capture.

The results from the ABC algorithm give us similar insights to what we obtained from a full parameter scan (the results in Fig. 3E). However, it is less computationally intensive. We conclude from the results here that good fits to the data can be obtained using the two-ODE model using Arrhenius scaling for the four main parameters. The parameter sets that best capture the scaling in the data and the upper thermal limit have activation energies for Cdc25 and Wee1 (activation/inactivation) that are close and different values for the production and degradation activation energies.

E. Fitting embryo data with the five-ODE mass action model. The five-ODE mass action model has ten different activation energies. A full parameter scan is unfeasible here, but the ABC algorithm can still be used. We also used 200 particles and let the algorithm run for 15 generations. Prior distributions were uniform on $[0, 150]$ kJ/mol for all activation energies. We also included a period scaling to avoid every temperature-period curve going through the same value at 18 degrees, as explained above. This value was close to one for all sampled parameter sets.

Good fits can generally be obtained using this model, although there are not so many parameter sets that can capture the bend upwards for high temperatures Fig. S5C. We are not entirely sure whether this is unexpected or not: in general more parameters to vary increases the possibility of an accurate fit, and ten parameters is already quite a lot. On the other hand, the oscillations in this system are quite sensitive to changes in the rate constants, making a good fit more difficult. We did observe that the sets of activation energies that provide a good fit are more localized in parameter space, whereas for the two-ODE model there were quite broad areas that gave a good fit.

The output of the algorithm is now a set of parameters which represents a probability distribution in ten-dimensional space. This is not so easy to visualize. We can however look at some summary statistics of this distribution, as in Fig. S5D. This figure shows the marginal distribution of the different activation energies and the pairwise correlations between them. Some of the pairs with high correlations correspond to antagonistic rates, such as $k_{p,g}$ and $k_{d,g}$. This suggests that the parameter sets that provide a good fit have more or less equal activation energies for the faster reactions, forward and backward, and that the thermal limits are generated by an imbalance between the E_a of production and degradation rates. This is a tentative conclusion, however, since the complete distribution of the distribution of all 10 rates is not completely captured by looking only at pairwise correlations and marginal distributions.

Supplementary Note 4: Fitting rate measurements of individual regulatory processes

We describe how the rates for cyclin B synthesis, CDK1 activity, PP2A activity and APC/C activity were fitted from the times series to obtain Fig. 6. For the linear fit we used the numpy function `polyfit`, and for the nonlinear fits we used the function

`curve_fit` from the `scipy.optimize` package.

A. Cyclin B synthesis rates. Fig. S13A-B shows a representative Western blot. By calculating the integrated density for each band and subtracting the background using FIJI, we obtain the intensity for each time point. This value is then divided by the average intensity for a CSF extract, to obtain the normalized intensities as shown in Fig. S13C. To obtain the cyclin accumulation rates, we next fit the slopes for each of the cycles, e.g. for the points indicated in S13C. The obtained results for the cyclin accumulation rates k_s from all Western blots, obtained from three independent extracts, are summarized in Fig. S7A (left).

B. Cdk1 rates. The time series from which we derive the rates are shown in Fig. S14. We fit a function of the form

$$y = at + b$$

to the time series, and save a as the resulting rate. We do this for every replicate, giving us multiple rate measurements per temperature. We used the non-P32 decay adjusted rates.

C. PP2A rates. The time series from which we derive the rates are shown in Fig. S15. We fit a function of the form

$$y = A(1 - e^{-kt})$$

to the time series. The rate is determined as the derivative of this function at $t = 0$, i.e. kA.

D. APC rates. The time series from which we derive the rates are shown in Fig. S16. We fit a function of the form

$$y = Ae^{-kt} + B$$

to the time series where we impose that $A, B, k > 0$. The rate we save is $-k$ and we only do this for the experiments label 'Mitotic'. We only fit this function form on times larger than or equal to 10 minutes, because the exponential decay does not start in the beginning.

Supplementary Note 5: Determination of the average cycle of CDK1 activity

In Fig. 5, we show an average cycle shape obtained from extracts. Here we explain how we obtained this. This is also illustrated in Fig. S11. For each temperature, we have a set of time series measurements (t_i, u_i), where u_i is the measurement of CDK activity (FRET ratio). We then process these data as follows:

1. We start with the values of time and CDK1 activity (t_i, u_i) for $i_1 \leq i \leq i_2$. Here i_1 and i_2 are the indices corresponding to peaks in the CDK signal.
2. We rescale time to the interval $[0,1]$: set $\tilde{t}_i = (t_i - t_{i_1})/(t_{i_2} - t_{i_1})$ and we shift the values of u vertically by subtracting the mean: $\tilde{u}_i = u_i - \bar{u}_i$.
3. We interpolate the values of $\tilde{u}$ at 100 evenly spaced time points in the interval [0,1] (excluding 1), obtaining a new time series $(\hat{t}_i, \hat{u}_i)$, $i = 1 \ldots 100$ with $t_1 = 0$ and $t_{100} = 1$.
4. We do this for all the time series at a given temperature, giving us $(t_{k,i}, u_{k,i})$ where k indexes the different time series (droplets). The average shape of the time series is then obtained by taking, for each i, the median of the values of u. The resulting time series is (t_i, U_i) with $U_i = \text{median}_{\text{over } k}\, u_{k,i}$

Supplementary Note 6: Direct estimation of the cyclin production rate scaling from extract time series

In Fig. 5B, we plot estimates of the scaling of k_s with temperature obtained directly from the time series. These measurements are the slopes of the increasing part of the CDK activity. We use a heuristic algorithm to determine these slopes automatically.

The time series mostly have a slow linear increase and then a fast jump. We want to capture the slope of the linear increase only. To detect the interval of this linear increase, we do the following. The idea behind this is shown in Fig. S12.

1. We start from one cycle of CDK activity with values (t_i, u_i) with $i_1 \leq i < i_2$ (as in Supp Note 5).
2. Let $i_m = \text{argmin}_i\, u_i$, the index of the minimal value of the u-values.
3. For j in $i_m + 2 \ldots i_2$, we determine the slope of a linear fit on the points $(t_i, u_i), i = i_m \ldots j$. In other words, we start from the first three points starting at i_m, and then always add one more point to the right until we hit the last point of the cycle. For each of these intervals, we obtain a slope a.
4. This yields a set of slopes a_j. We then look at this array starting from its last element, i.e. we have a_0 be the slope obtained fitting from i_m to the last point of the cycle, a_1 from i_m to the one-to-last point, etc.
5. We determine $r_j = (a_j - a_{j-1})/a_j$, the relative errors in this array of slope (j starting from 1).
6. Reasoning that first, r_j will be large because at the end of the cycle there are large variations, and that for points on the linearly increasing part of the cycle the relative errors do not change much, we fix the final slope as the one corresponding to the first relative minimum of the r_j (we exclude the first two r_j to avoid detecting the very first point as a minimum).

Supplemental Figures

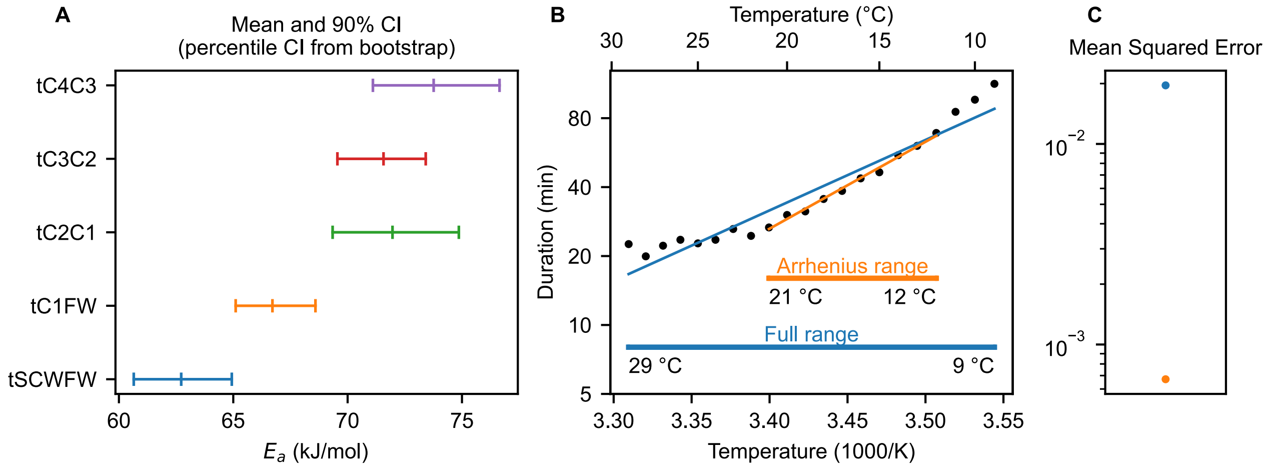

Fig. S1: **Cell division timing in early Xenopus laevis embryos does not scale Arrhenius over the whole temperature range** A. In Fig. 1C, we show the duration of several early developmental periods in function of temperature in the range $[T_{\text{min}} = 9°C, T_{\text{max}} = 29°C]$, with the apparent activation energy as obtained by an Arrhenius fit between 12°C and 21°C in Fig. 1D. Bootstrapping provides a probability distribution for the apparent activation energies (Fig. 1E). Here, we show the mean and 90% confidence interval (CI) for comparison, illustrating the statistical significance of the activation energy differences. B. Cleavage cycle duration in function of temperature for the second to fourth cell cycle in the range $[T_{\text{min}} = 9C, T_{\text{max}} = 29°C]$ for *Xenopus laevis*. Optimal fits using single exponential Arrhenius (SE) are shown in two different temperature ranges: from 12°C and 21°C (orange), and the whole temperature range (blue). The mean square error (MSE) is much higher over the whole temperature range than within the selected range (panel C), indicating that the Arrhenius equation does not fit the data well over the whole measured data range.

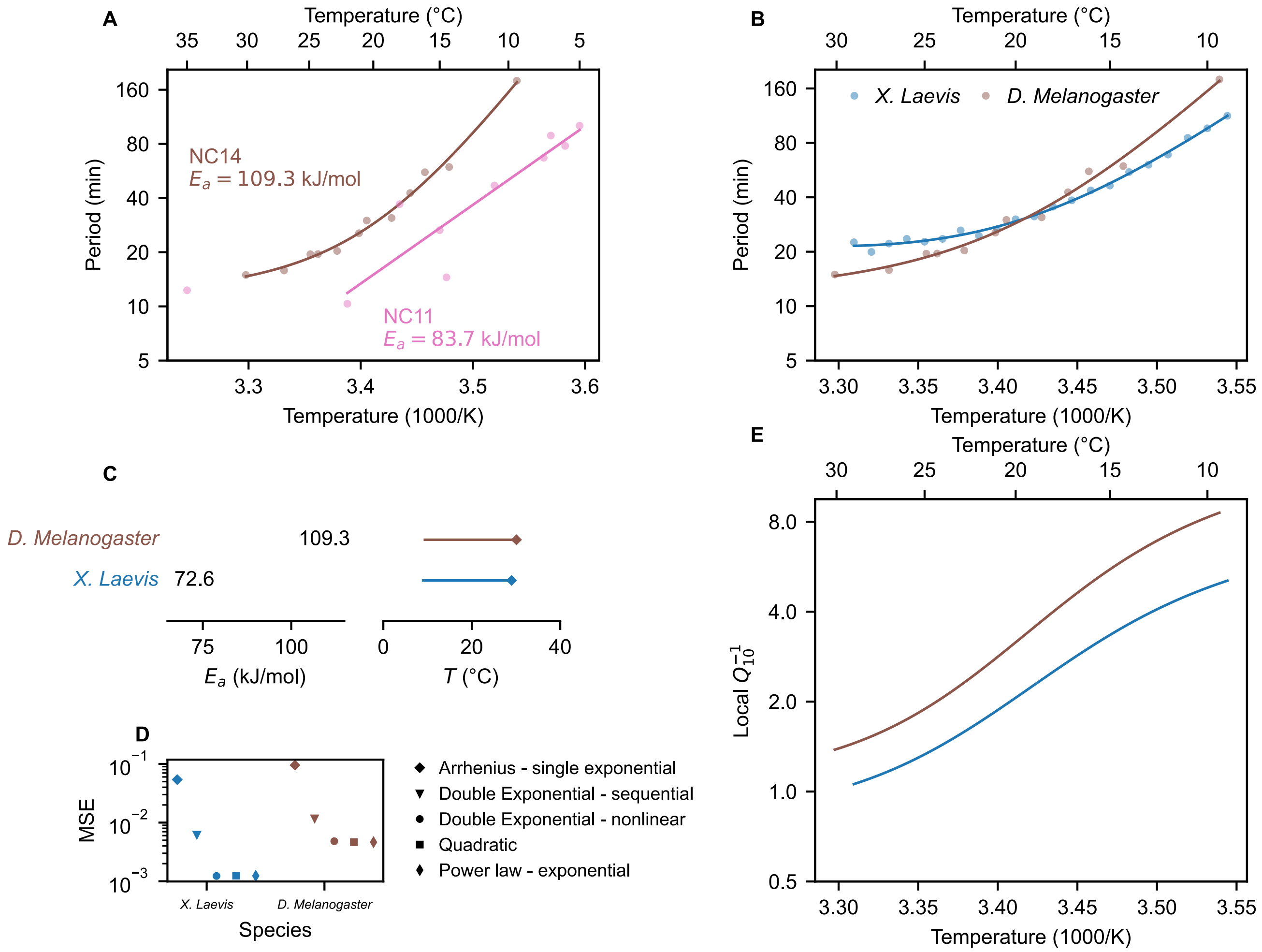

Fig. S2: **Temperature scaling of embryonic processes in *Drosphila melanogaster*** A. Median cleavage period in function of temperature for the eleventh and thirteenth cell cycle *D. melanogaster* (32, 33). Optimal fits using a double exponential (DE) function are overlayed. B. Median cleavage period in function of temperature for the second to fourth cell cycle (all pooled) in *X. laevis* (this work), and for the eleventh and fourteenth cell cycle *D. melanogaster* (32), together with double exponential (DE) best fits. C.Activation energy, minimal and maximal temperature for *X. laevis* and *D. melanogaster*, corresponding to the curves in panel B. D. Comparison of the mean squared error (MSE) for fits with different functional forms, as in Fig. **??**G. E. Using the best DE fit, the local Q_{10} value is plotted in function of temperature.

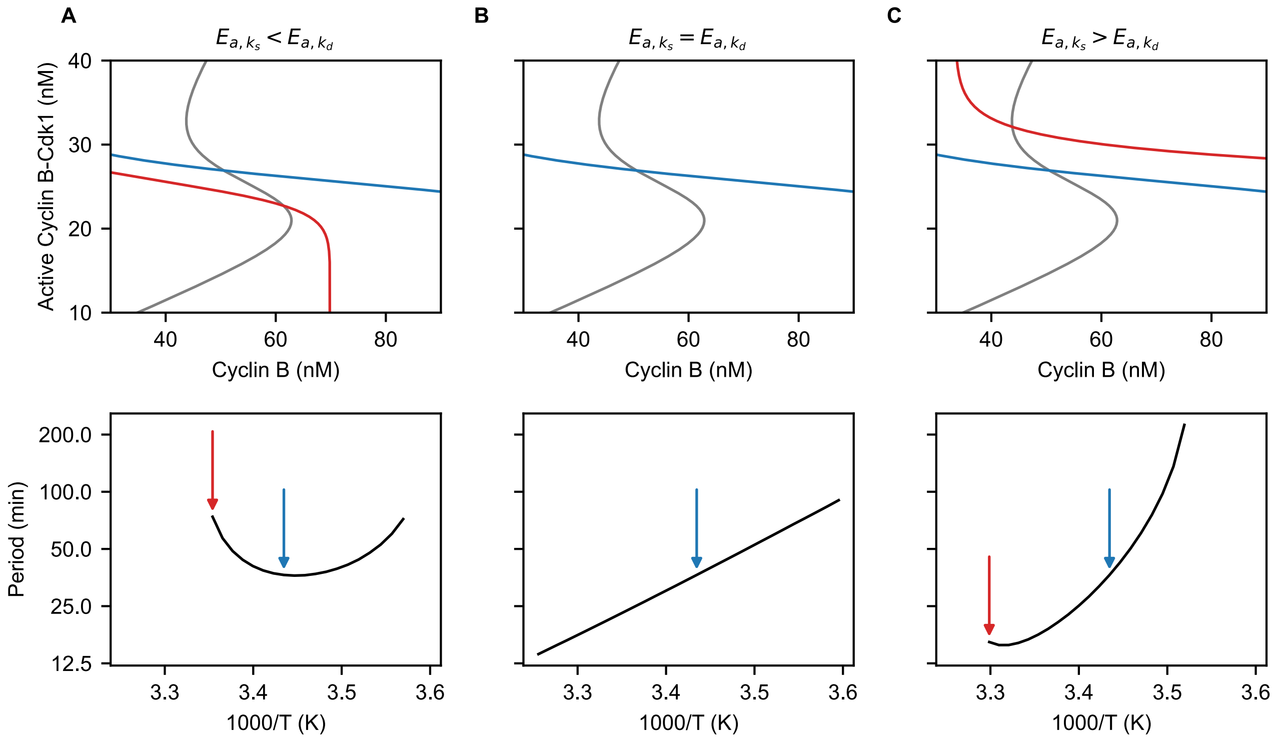

Fig. S3: **Temperature dependence of nullclines in the phase plane.** In the two-ODE cell cycle model, thermal limits are determined by intersection of nullclines. Depending on the relative size of E_{a,k_s} and E_{a,k_d}, the non-S-shaped nullcline (red/blue) shifts upward or downward with rising temperatures. When the intersection of the nullclines lies on the upper or lower branch of the S-shaped nullcline (gray), oscillations cease to exist. A. The system ends up in a low-activity state at high temperatures. B. The second nullcline is temperature independent. There is no thermal limit. C. The system is stuck in a high-activity state at high temperatures.

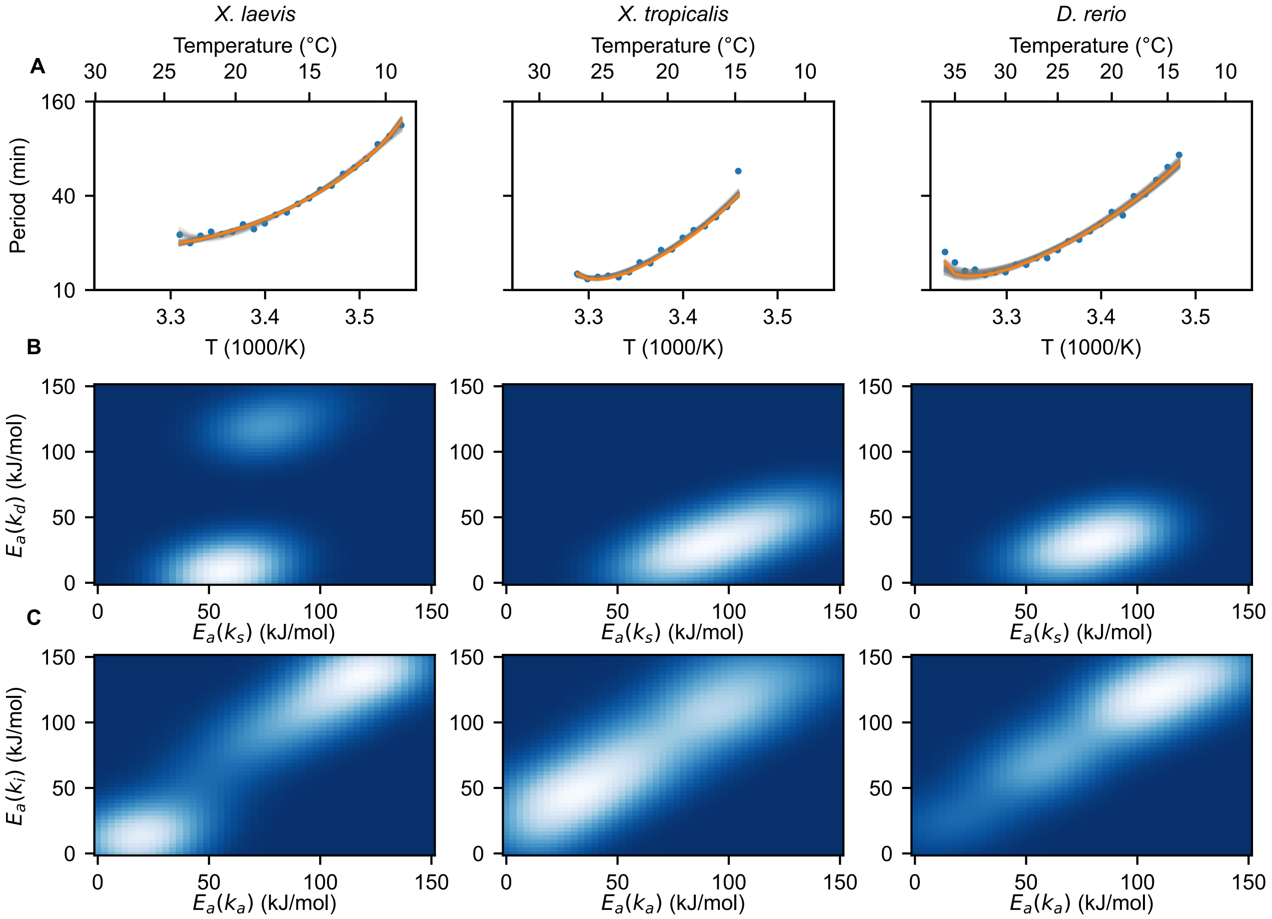

Fig. S4: **Optimal fits of the two-ODE model to the measured data using the ABC method** A. Resulting fits from the ABC algorithm for the two-ODE model. Gray lines show the 200 resulting parameter sets, with more transparent lines corresponding to lower weighted points. The orange line is the best fit (smallest distance). B. Projection of the four-dimensional probability density onto the $(E_a(k_s)$, $E_a(k_d))$ -plane. B. Projection onto the $(E_a(k_a)$, $E_a(k_i))$- plane. The heatmaps were constructed from 200 weighted samples and smoothed with a Gaussian kernel.

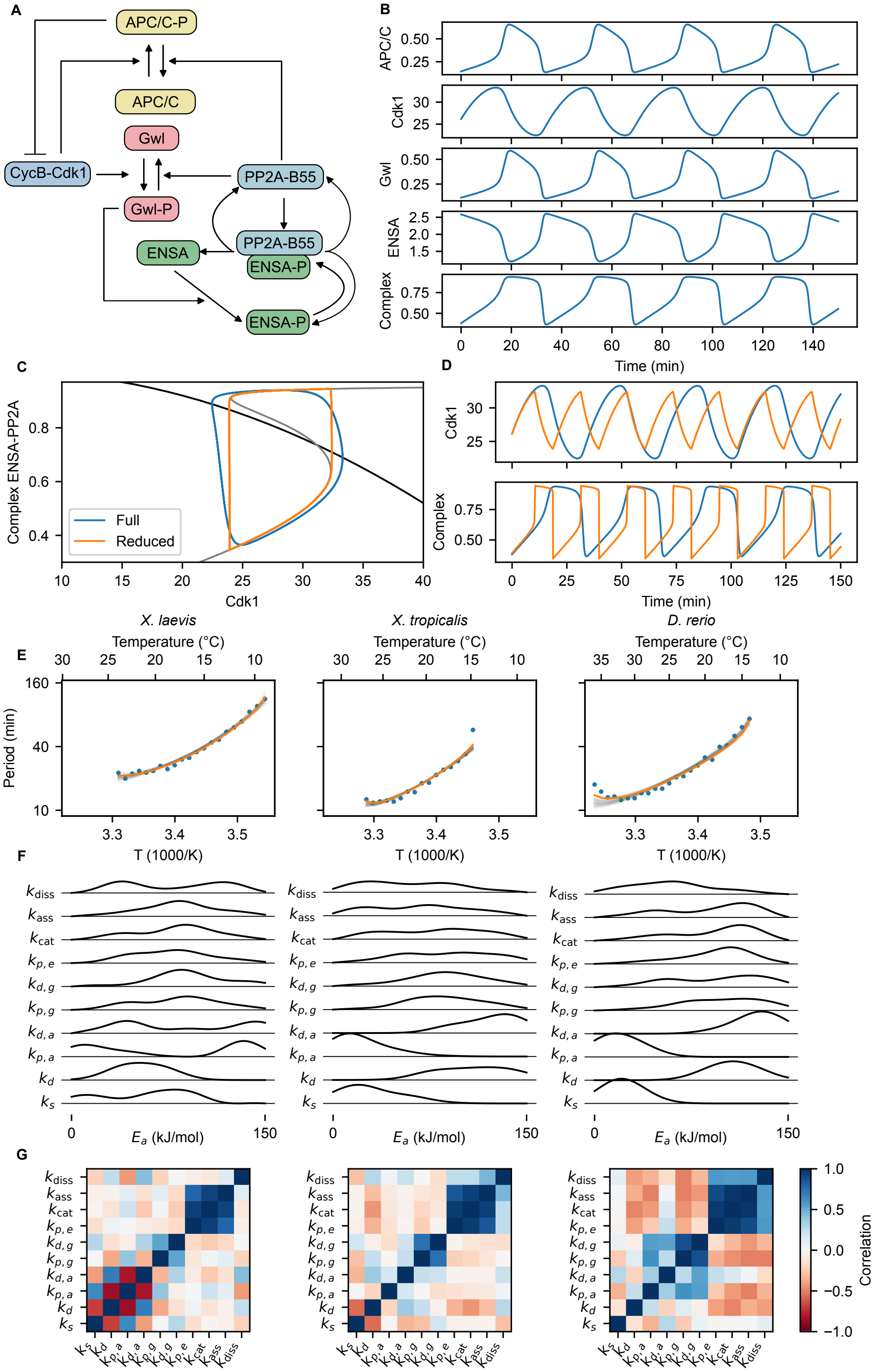

Fig. S5: **The five-ODE mass action model and fits to the measured data using ABC method** A. The interaction diagram for the five-ODE mass action model. B. Representative time series of a simulation of the 5-equation model. C. Phase plane of the reduced two-ODE model and projection of the five-ODE model onto this plane. Nullclines are shown in black and gray, the blue limit cycle is the projection of the oscillation of the five-ODE system and the red limit cycle is the one in the two-ODE system. D. Time series of corresponding variables in the full (blue) and reduced (orange) model. E. Resulting fits from the ABC algorithm for the mass action model. Gray lines show the 200 resulting parameter sets, with more transparent lines corresponding to lower-weighted points. The orange line is the best fit (smallest distance). F. Results of the ABC algorithm for the mass-action model. Marginal distributions of the different activation energies (smoothed using a Gaussian kernel). G. Pairwise correlation of the activation energies of the different rates computed from the result of the ABC algorithm.

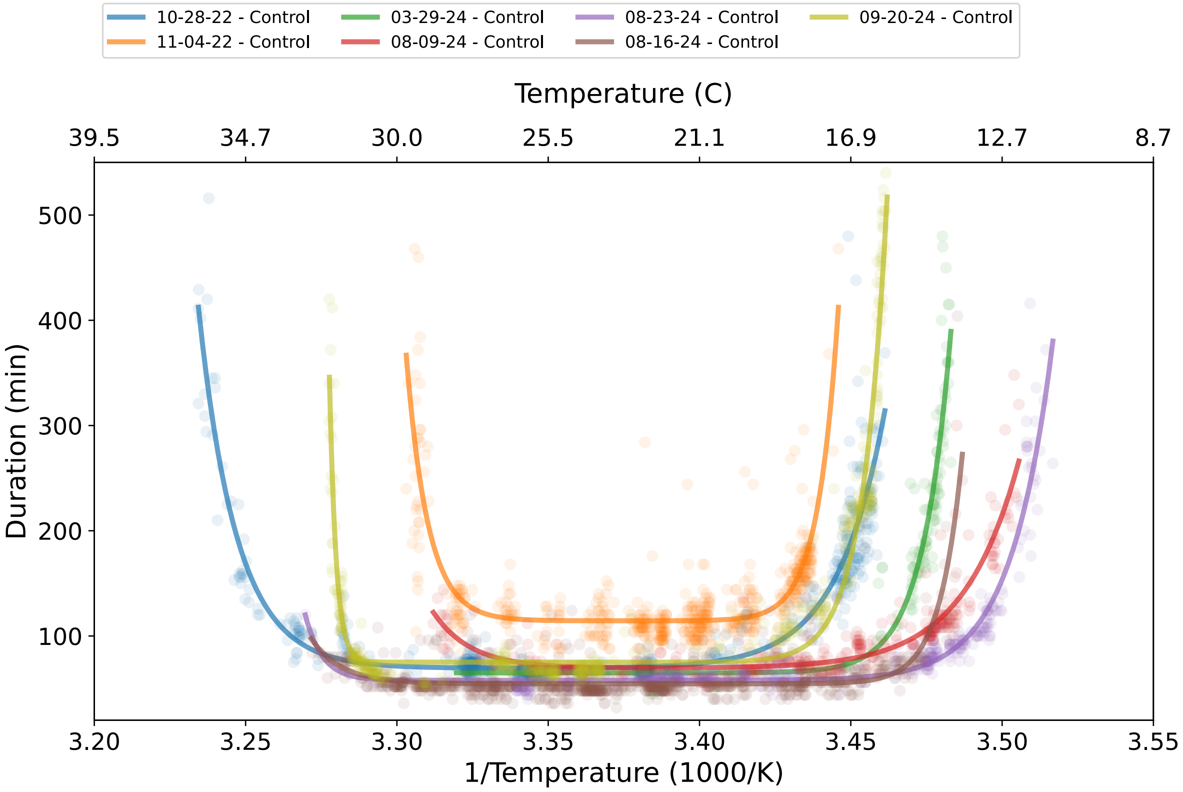

Fig. S6: **Comparison of temperature response across different replicates** Duration of the first detected cycle as a function of temperature for different biological replicates (different frogs and different experimental days). Dots represent individual droplet cycles and solid line corresponds to a double-exponential fit of the data.

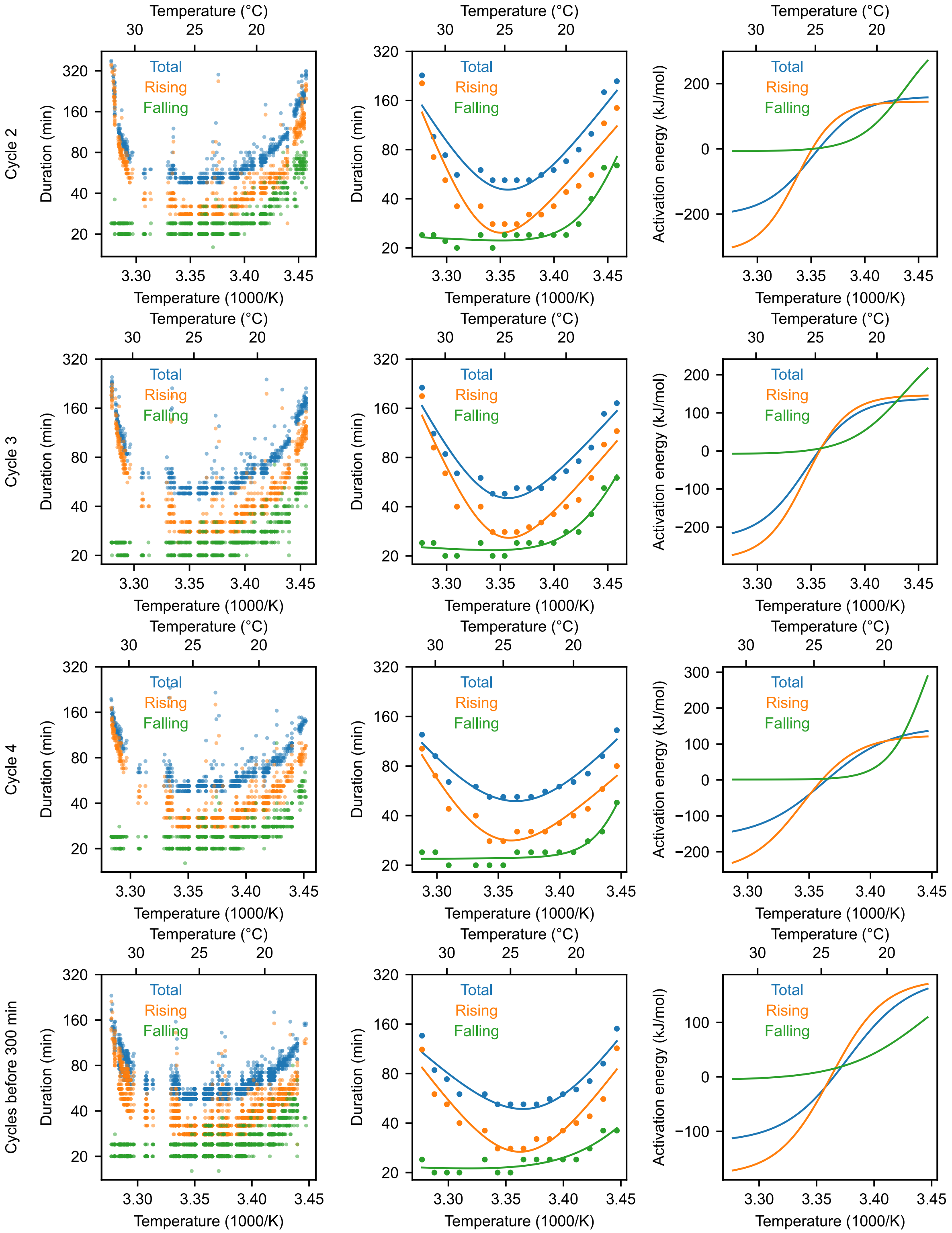

Fig. S7: **Fitting the duration of the total cell cycle, rising phase, and falling phase.** Analogous to Fig. 4C-E: left the raw data, middle the median per rounded temperature with double exponential fit, right the local E_a computed from the double exponential fit. Three first rows are the data for cycles 2, 3, 4 separately. The bottom row shows the plots combining all the cycles that occur in the first 300 minutes of the experiment.

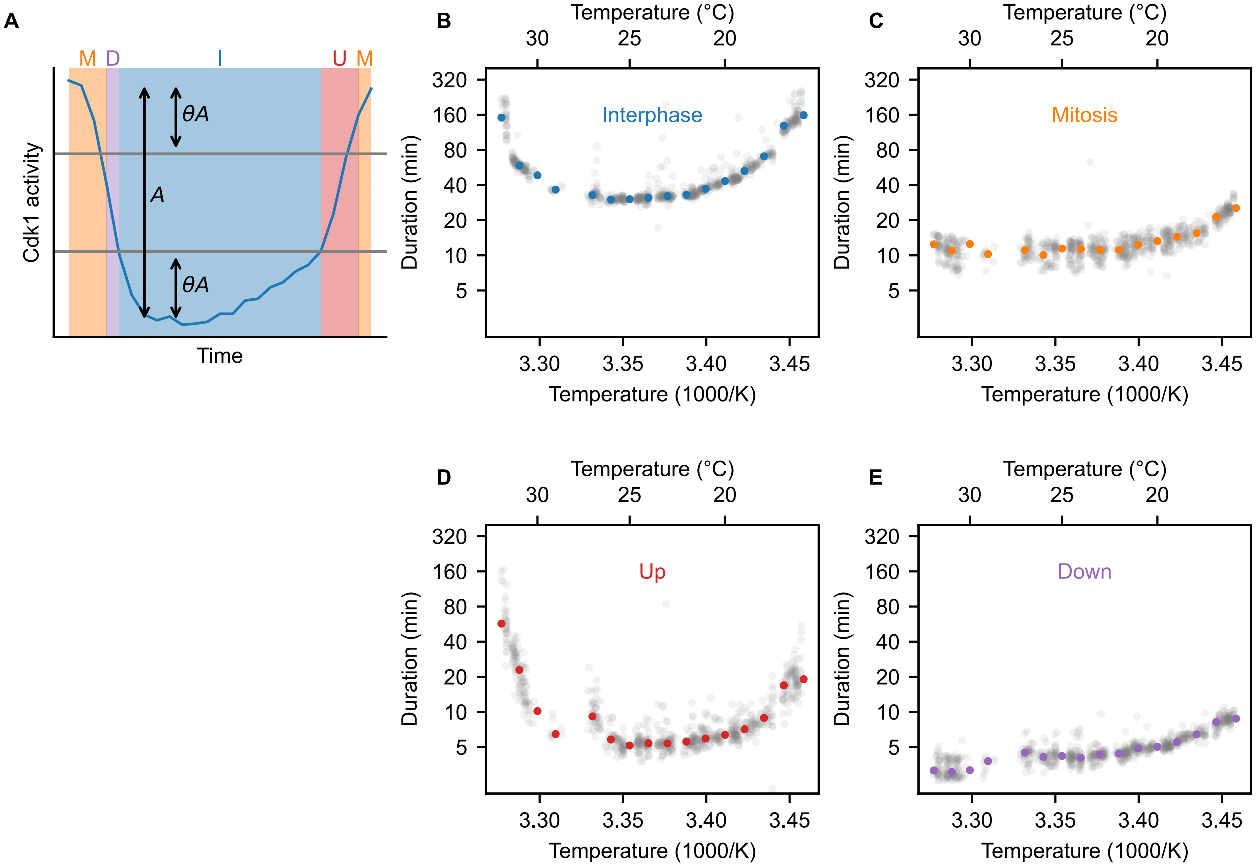

Fig. S8: **Scaling of four parts of the cycle in extracts.** A. Diagram of the method for determining the duration of four parts of the cycle. After determining the minimal and maximal value of the cycle, we determine the amplitude A and two threshold values determined by θ, which we take to be 0.3. Duration of interphase is the time the cycle is below the lower threshold, mitosis is the time the cycle spends above the higher one, and up and down times are the times it spends in between. B-E Duration of these four parts as function of temperature. Light gray dots are datapoints from all droplets, colored dots show the median duration per temperature.

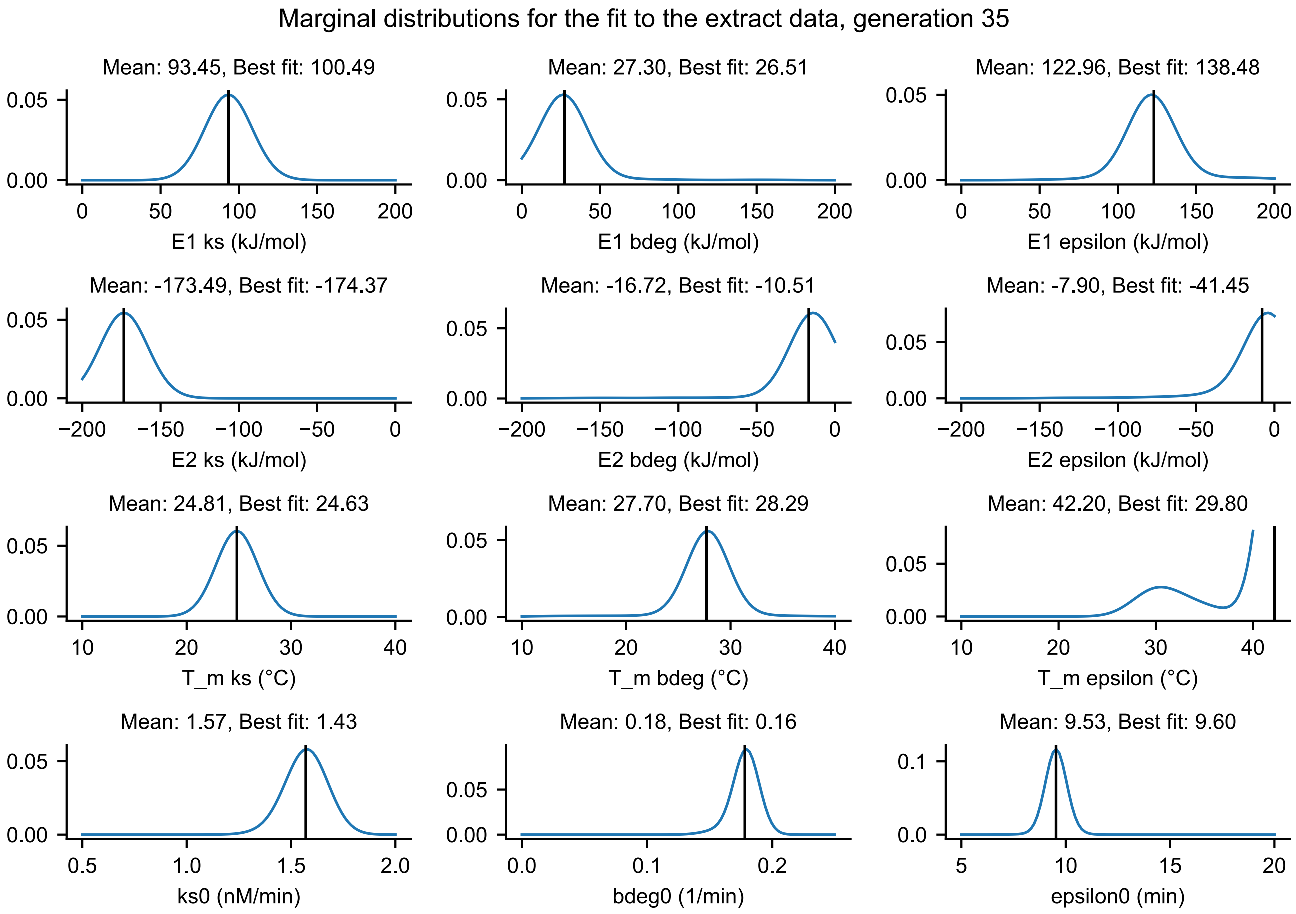

Fig. S9: Marginal distributions of the parameter sets. Corresponds to the temperature scaling of parameters shown in Fig. 4.

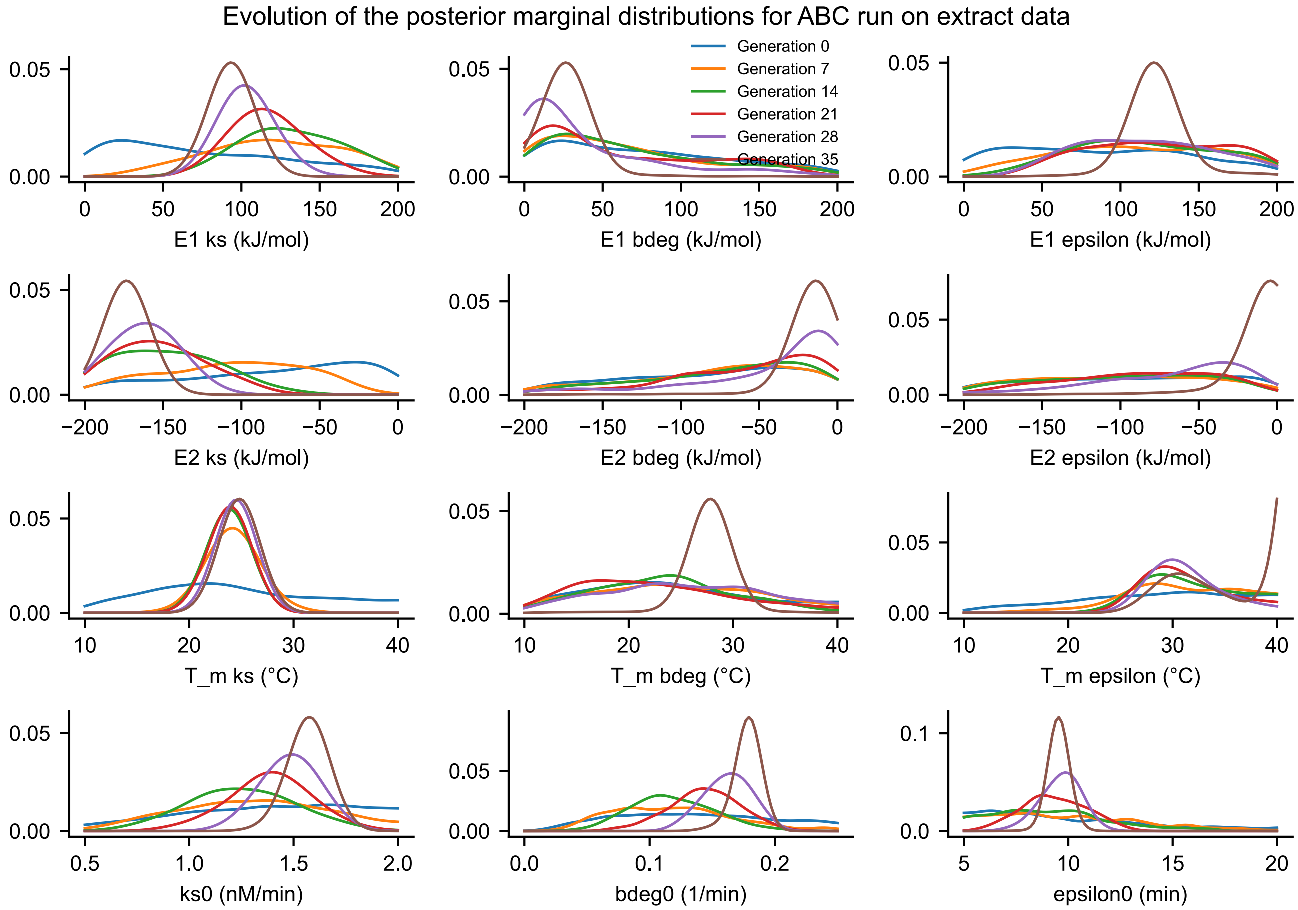

Fig. S10: Evolution of the marginal distributions of the parameter sets over the course of the ABC algorithm. Corresponds to the temperature scaling of parameters shown in Fig. 4.

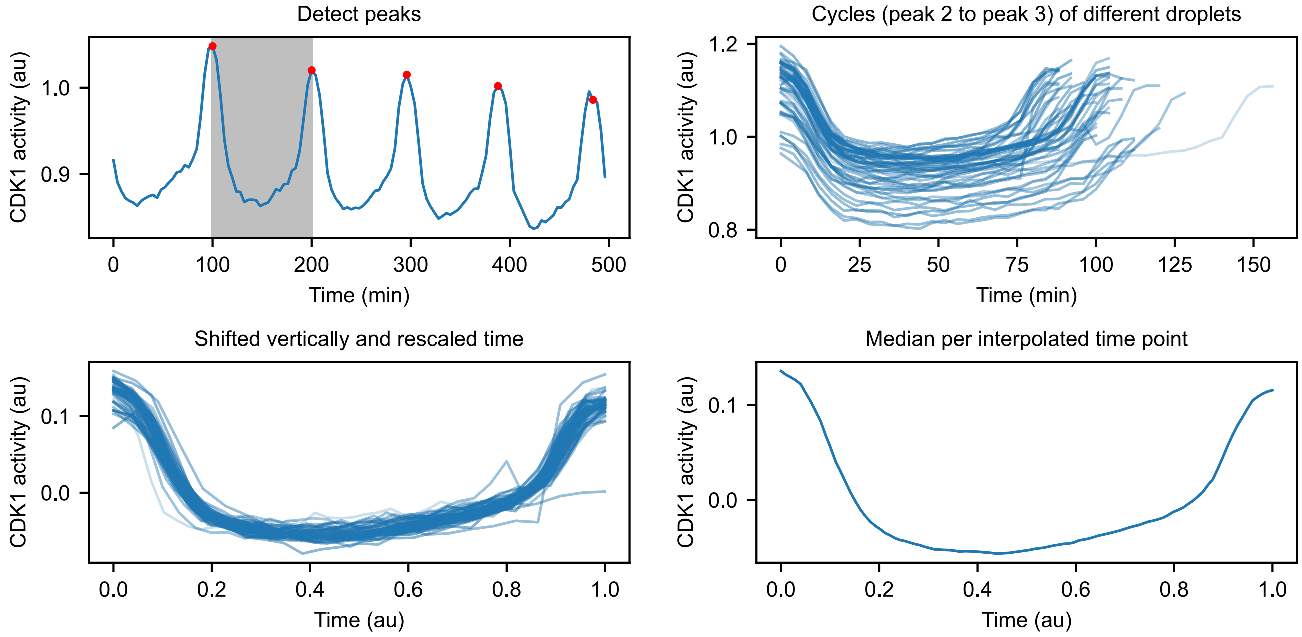

Fig. S11: Determination of the average cycle shape for CDK1 activity per temperature, in this case for $T = 22$ degrees Celsius. the bottom right one shows the final average cycle.

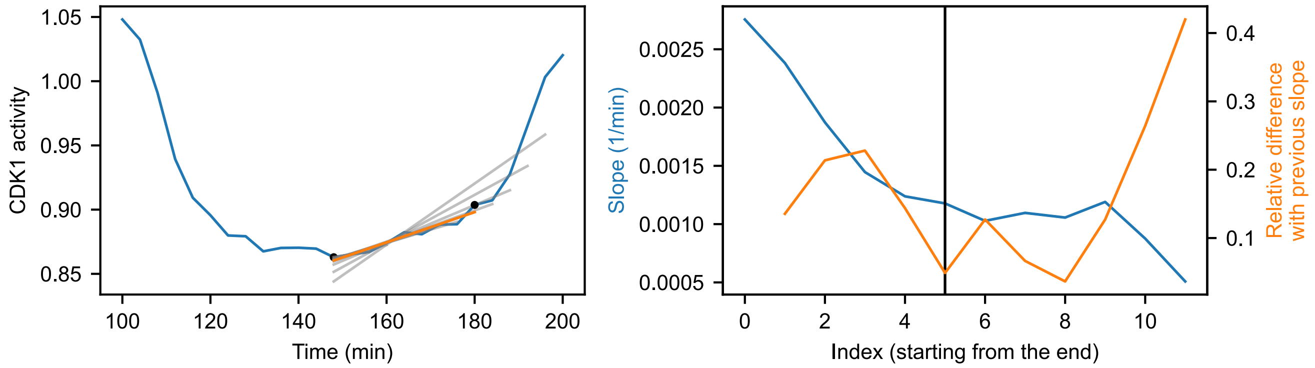

Fig. S12: Determination of the cyclin synthesis rate k_s from the time series.

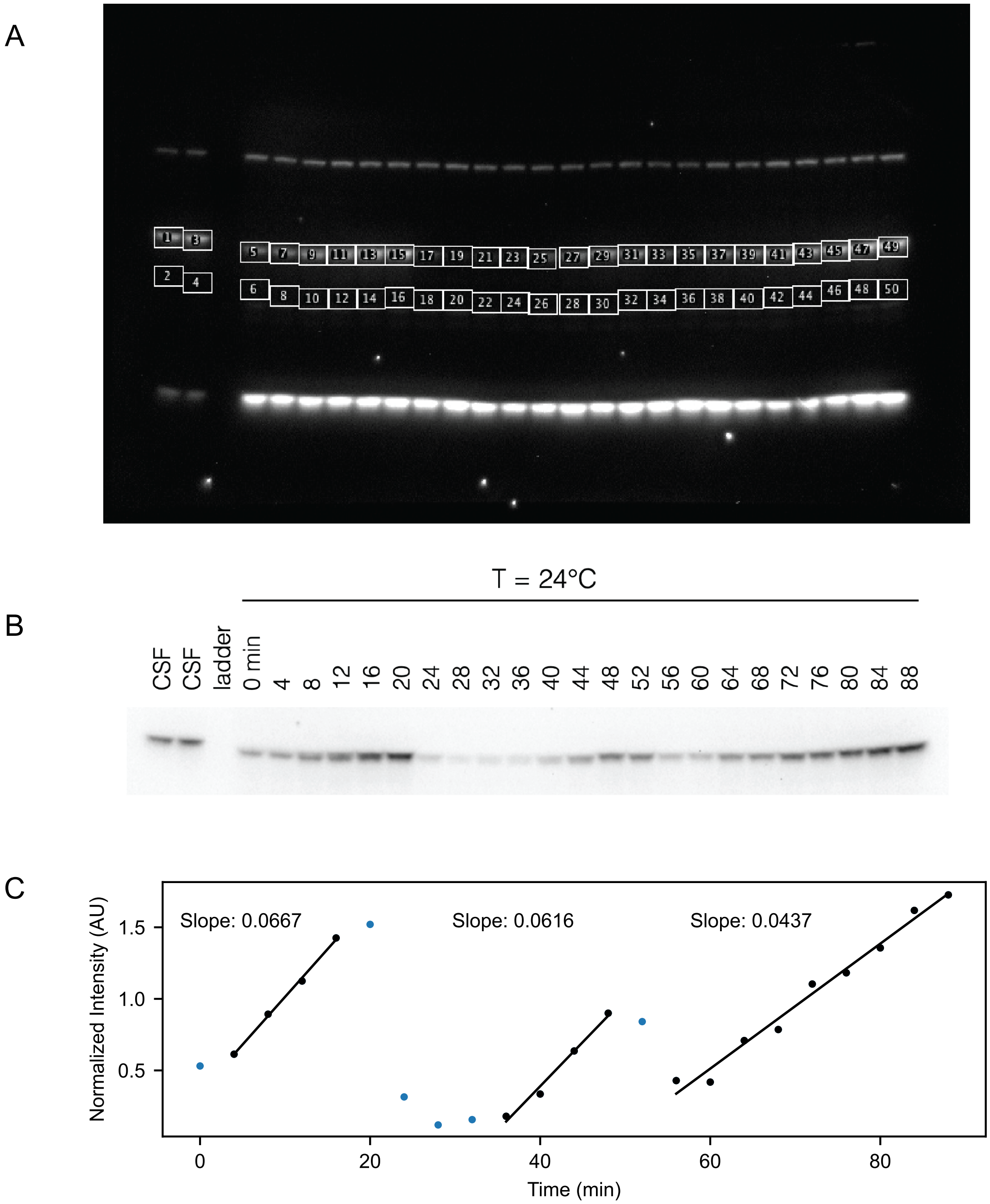

Fig. S13: **Measuring cyclin synthesis rates using quantitative Western blotting**. A. Time course of a representative Western blot using anti-cyclin B2 antibody on a cycling frog egg extract. B. Selected region of same Western blot as in A. C. Quantification of the Western blot by calculating the integrated density for each band and subtracting the background using FIJI, we obtain the intensity for each time point. This value is then divided by the average intensity for a CSF extract, to obtain the normalized intensities. To obtain the cyclin synthesis rates, we next fit the slopes for each of the cycles for the points indicated in black.

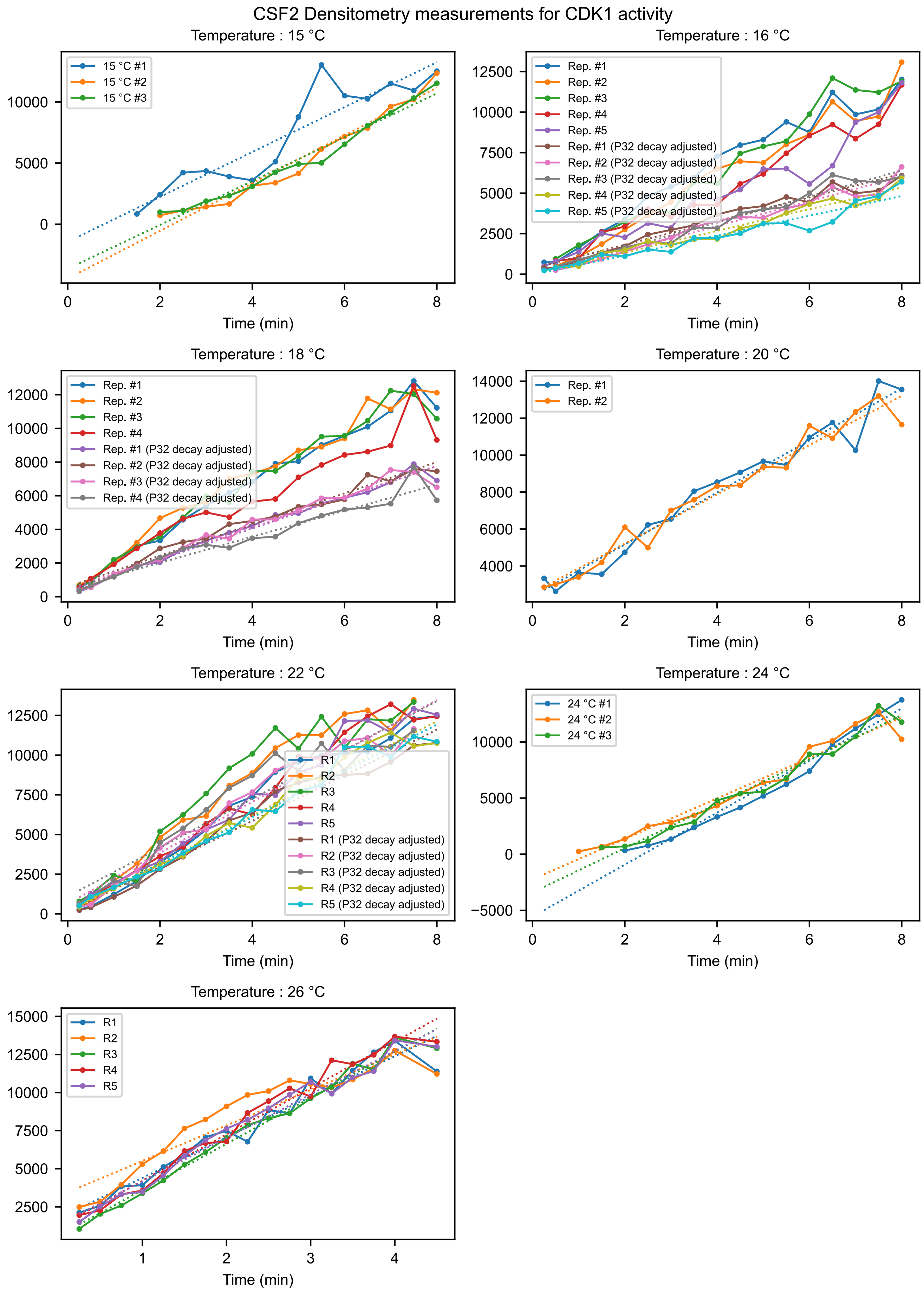

Fig. S14: Time series for CDK1 activity.

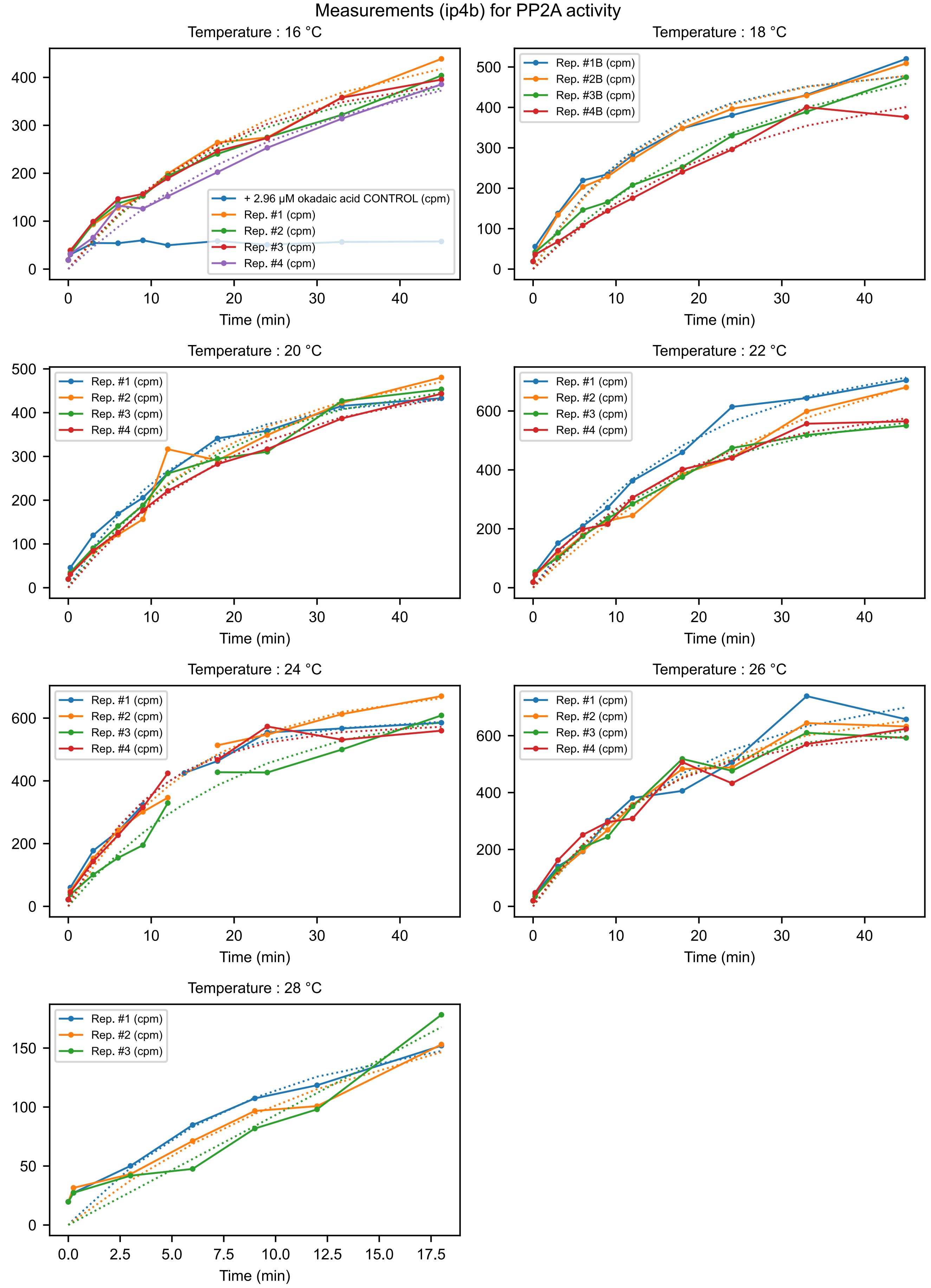

Fig. S15: Time series for PP2A activity.

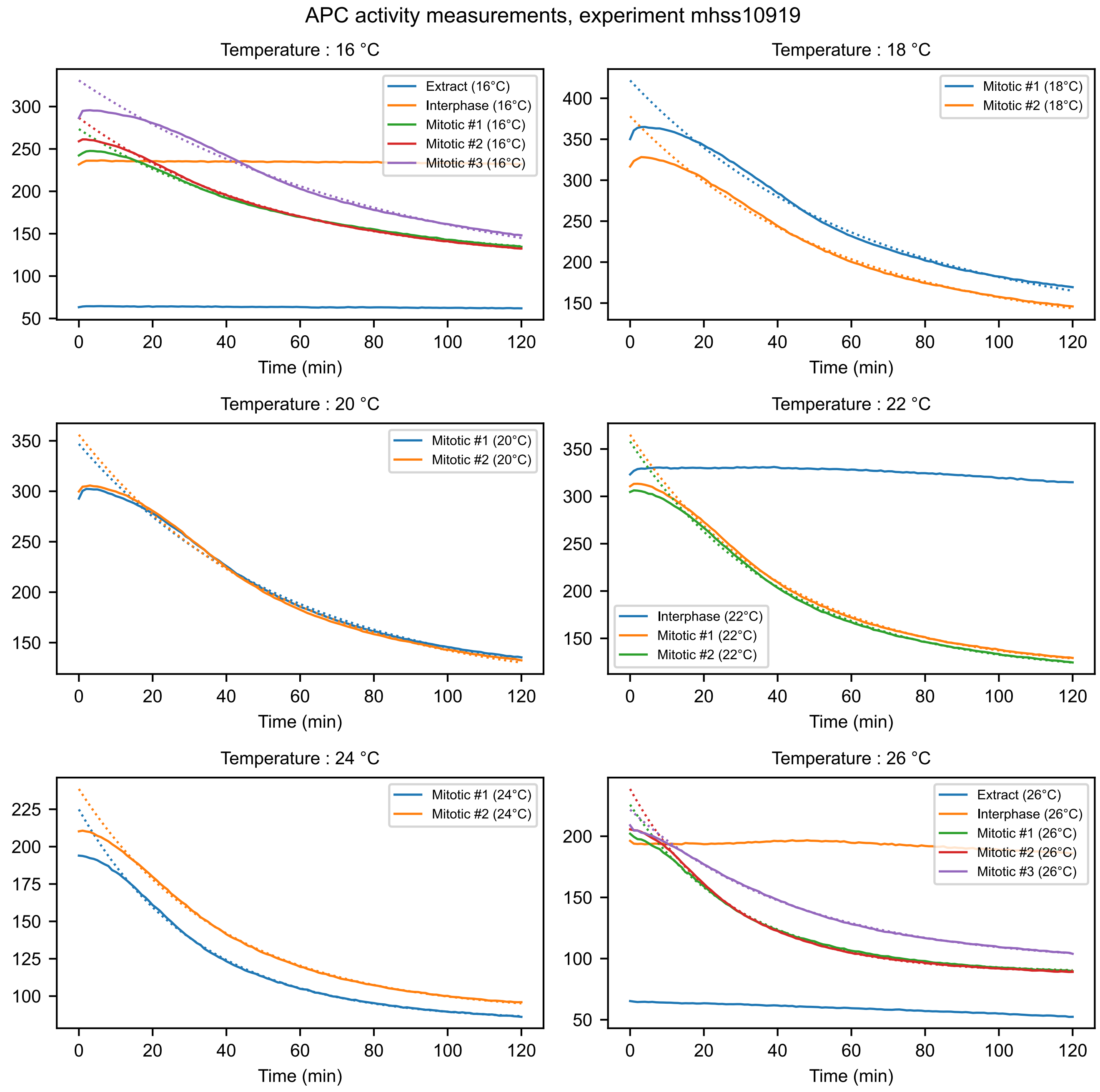

Fig. S16: Time series for APC activity.
